# A Thermal State Field Forecasting Framework for Intelligent Ventilation Decision-Making in Granary Based on a Hierarchical TCN-Mamba Network

**DOI:** 10.3390/s26175362

**Published:** 2026-08-25

**Authors:** Hongwei Zhang, Jingyu Yang, Yating Zhu, Zhuo Gao, Enze Ren, Bixian Li

**Affiliations:** School of Electronic Information Engineering, Anhui University, Hefei 230601, China

**Keywords:** grain storage, temperature forecasting, distributed temperature sensing, thermal state field, Mamba, temporal convolutional network, spatial attention, event-triggered ventilation decision support

## Abstract

Temperature forecasting from distributed temperature sensing supports thermal characterization and ventilation decisions in grain storage. However, existing studies mainly focus on individual sensor points and are insufficient to describe spatial thermal evolution. This study proposes a thermal state field forecasting framework integrating a Temporal Convolutional Network and Mamba. Sensor observations are aggregated into spatial blocks to construct average temperature, temperature variance, and three-dimensional temperature gradients. Spatial attention captures local correlations, while the hierarchical TCN and Mamba encoder and Cross-Scale Trend Attention extract multi-scale temporal features and long-range evolution patterns. Across all 30 spatial blocks, the proposed model achieved RMSE values of 0.2988 ± 0.0374, 0.5573 ± 0.0112, 0.0380 ± 0.0009, 0.0442 ± 0.0031, and 0.0788 ± 0.0050, and MAE values of 0.2591 ± 0.0330, 0.4270 ± 0.0093, 0.0344 ± 0.0008, 0.0379 ± 0.0031, and 0.0684 ± 0.0053 for average temperature, temperature variance, and gradients in the X, Y, and Z directions, respectively. Based on the predicted thermal state field, an illustrative forecast-driven decision-support framework provides event-triggered ventilation decision support and regional priority recommendations. The decision analysis further illustrates the relationship between predicted thermal evolution and ventilation timing and regional prioritization under predefined rules.

## 1. Introduction

Grain storage plays a critical role in ensuring national food security [1,2], and thermal regulation during storage is an important factor affecting grain quality and storage safety [3,4]. With the continuous expansion of modern granaries and extended storage periods, thermal evolution within grain piles is influenced by multiple factors, including external climatic changes, internal heat transfer, and airflow conditions [5,6,7]. These coupled factors may result in localized heat accumulation. Persistent heat accumulation can accelerate grain quality deterioration and increase the risks of mold growth and insect infestation, thereby causing grain storage losses [8]. Therefore, accurately characterizing the evolution of thermal states within granaries and implementing proactive measures in regions with potential thermal risks remain important research objectives in the development of intelligent granaries.

In current grain storage management systems, mechanical ventilation is one of the most widely used approaches for temperature regulation [9,10,11,12]. Proper ventilation can reduce grain temperature and suppress localized thermal anomalies. However, practical ventilation decisions are still mainly based on manual experience or fixed temperature thresholds [13,14,15]. Typically, fans are activated when temperatures at monitoring points exceed preset thresholds and stopped when temperatures fall below these limits. Although this strategy is simple and easy to implement, it is essentially a reactive control approach. Thermal anomalies are often identified only after heat accumulation has developed, resulting in delayed intervention and potential risk propagation to surrounding areas. Moreover, the thermal environment within granaries exhibits strong spatial heterogeneity [16], with different regions showing distinct thermal evolution patterns. Uniform ventilation may lead to unnecessary operation in low-risk areas while failing to provide timely regulation for regions requiring priority treatment. Therefore, monitoring-based passive control strategies are insufficient to meet the requirements of modern granaries in terms of both storage safety and operational efficiency.

To improve the proactiveness and specificity of ventilation control, researchers have begun to integrate predictive technologies into grain management processes to identify potential risk areas before thermal anomalies develop and to formulate corresponding ventilation plans in advance. Against this background, precision ventilation [17,18] has gradually emerged as an important research direction in smart grain storage. Precision ventilation focuses not only on whether ventilation is necessary, but also on when ventilation should be performed and which areas require ventilation.

Accurate prediction of future thermal states provides an important basis for such precision ventilation decisions. Accordingly, substantial research has focused on granary temperature forecasting. Early studies primarily employed heat conduction equations, finite element models, and computational fluid dynamics models to describe heat transfer processes within grain piles. For example, Montross et al. [19] proposed a finite-element grain storage ecosystem model to simulate and analyze heat and mass transfer processes within grain piles by numerically solving the corresponding governing equations. However, such methods typically require numerous thermophysical parameters and boundary conditions, resulting in complex model construction and relatively high costs in practical applications. Subsequently, data-driven methods such as Support Vector Machines (SVMs), Random Forests (RFs), and Artificial Neural Networks (ANNs) have gradually been applied to temperature prediction tasks. Duan et al. [20] combined a support vector regression (SVR) model with meteorological data to predict grain pile temperatures by learning the relationships between historical grain temperatures and environmental factors. Wang [21] proposed a random forest (RF)-based temperature prediction model for ladle furnaces, in which multiple decision trees were integrated to learn complex nonlinear relationships from large-scale industrial process data. Juarez-Balderas et al. [22] developed an artificial neural network (ANN)-based model for predicting transformer hotspot temperatures by learning nonlinear feature relationships from historical operational data. In recent years, with the development of deep learning technologies, recurrent neural networks such as LSTM and GRU have also been widely applied to granary temperature forecasting, with promising predictive performance. Among these, Li et al. [23] developed an integrated deep learning-based intelligent grain storage solution for granary monitoring and risk early warning, using a 3DCNN-LSTM to achieve intelligent perception of the grain storage environment and anomaly early warning. Mao et al. [24] proposed a granary temperature prediction method based on a GRU deep fusion model, which exploits the temporal features of grain temperature to predict grain pile temperature. Subsequently, models such as Transformer [25], Informer [26], FEDformer [27], PatchTST [28], and iTransformer [29] have further advanced long-sequence prediction performance. Meanwhile, Mamba, which is based on state-space models, has demonstrated high computational efficiency and strong long-range dependency modeling capability in long-sequence modeling tasks [30,31] and has gradually attracted increasing research attention.

Despite the progress in prediction accuracy, existing studies mainly focus on point-level temperature forecasting. Measurements from discrete sensor locations provide only localized thermal information and are insufficient to describe the spatial evolution of thermal states within grain piles. Although multiple monitoring points have been used to analyze overall temperature distributions [32,33], independently forecasting numerous sensor locations increases model complexity and remains weakly connected to practical ventilation regions. Moreover, the thermal evolution of grain piles is a complex spatiotemporal process [34], and temperature information alone cannot comprehensively characterize regional thermal conditions. Thermal characteristics such as temperature distribution, spatial variability, and heat transfer trends provide complementary information for describing regional thermal conditions. Therefore, a regional thermal state field forecasting framework that integrates spatial correlations and multi-dimensional thermal characteristics is required to support intelligent ventilation decision-making.

This study develops a thermal state field forecasting framework for intelligent ventilation decision-making in granaries. The proposed framework aggregates distributed sensing data into spatial blocks and characterizes regional thermal conditions using multiple thermal features, including average temperature, temperature variance, and three-dimensional temperature gradients. A Hierarchical TCN-Mamba network is developed to capture multi-scale temporal evolution patterns, where Spatial Neighborhood Attention is introduced to model spatial interactions among adjacent regions. Furthermore, a Cross-Scale Trend Attention module is incorporated to enhance long-term trend representation, and a residual delta prediction strategy is adopted to improve forecasting stability. Based on the predicted thermal state field, an illustrative forecast-driven event-triggered ventilation decision-support framework is formulated to identify regions with potential ventilation demand and provide corresponding region-level ventilation priority recommendations. The main contributions of this study are summarized as follows:

1. A thermal state field representation method is developed by aggregating distributed sensor measurements into spatial blocks. Multiple thermal characteristics, including average temperature, temperature variance, and three-dimensional temperature gradients, are integrated to characterize regional thermal evolution beyond conventional point-level temperature monitoring.
2. A Hierarchical TCN-Mamba network is developed to capture complex thermal evolution patterns. Spatial Neighborhood Attention is introduced to model interactions among adjacent regions, while Cross-Scale Trend Attention and residual delta prediction are incorporated to enhance long-term forecasting capability and stability.
3. An illustrative forecast-driven event-triggered ventilation decision-support framework is formulated based on the predicted thermal state field. The framework identifies regions with potential ventilation demand and provides region-level ventilation priority recommendations, thereby linking thermal state field forecasting with ventilation-oriented decision support.

The remainder of this paper is organized as follows. Section 2 describes the experimental dataset, spatial partitioning strategy, and thermal state feature construction method. Section 3 presents the proposed thermal state field forecasting framework, including spatial feature extraction, hierarchical temporal modeling, trend representation, and the event-triggered ventilation decision-support framework. Section 4 presents the experimental results, including comparative experiments, ablation studies, forecast-horizon error analysis, spatial thermal evolution analysis, event-triggered ventilation decision-support analysis, and parameter sensitivity analysis. Finally, Section 5 summarizes the main findings of this study and discusses potential directions for future research.

## 2. Data Processing and Analysis

### 2.1. Datasets

**Figure 1:**
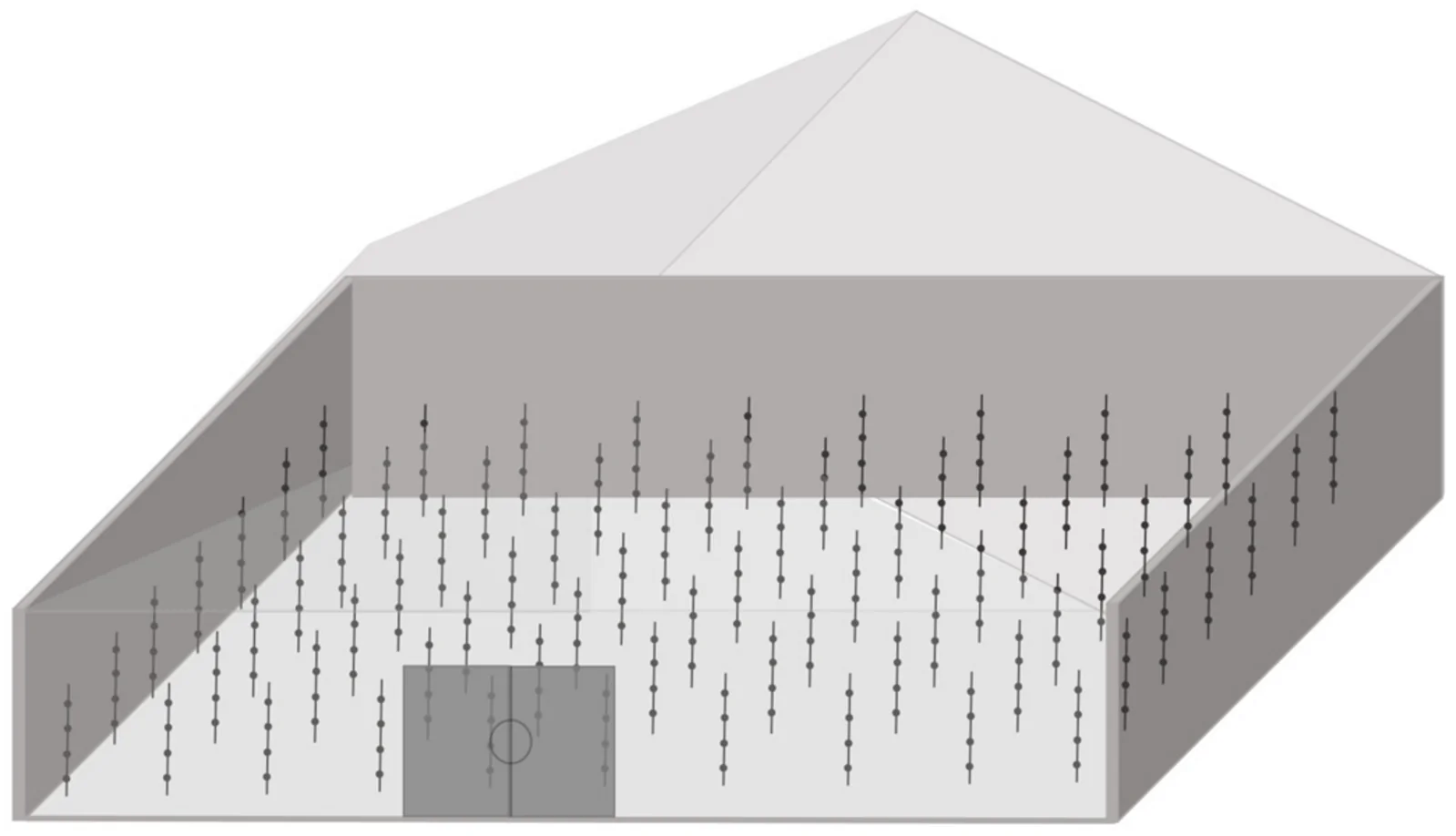
Layout of the Flat Grain Warehouse and Temperature Sensor Deployment. The vertical lines represent the sensor cables, and the dots indicate the locations of the temperature sensors.

The experimental data were obtained from a sensor monitoring system installed in a flat grain warehouse in the Jianghuai region of China. Temperature sensors were deployed inside the grain pile to continuously monitor internal temperature variations. To characterize the spatial distribution of thermal states within the grain pile, the sensors were uniformly arranged along the column, row, and height directions, forming a 10 × 7 × 4 three-dimensional monitoring networks comprising 280 monitoring nodes, as shown in Figure 1.

The sensor deployment covered both the central and peripheral regions of the grain pile, allowing temperature changes at different spatial locations to be recorded. In addition to internal temperature measurements, external meteorological variables, including outdoor air temperature and outdoor humidity, were collected synchronously during the storage period. The monitoring data were recorded daily at 14:00 from 4 August 2019 to 4 August 2021 and were arranged in chronological order to form a continuous daily time series. A summary of the dataset characteristics is provided in Table 1.

Before model training and evaluation, the raw monitoring data were processed through a quality control procedure. Missing values and abnormal observations caused by sensor fluctuations or acquisition errors were first identified and removed. The resulting gaps were then filled using temporal linear interpolation based on adjacent valid observations:

(1) $${\hat{x}}_{t}=\frac{x_{t-1}+x_{t+1}}{2}$$

where $x_{t-1}$ and $x_{t+1}$ denote the nearest valid observations immediately before and after time *t*, respectively, and ${\hat{x}}_{t}$ represents the interpolated value.

Given the differences in the range of temperature variations across different monitoring locations, the data were standardized using the Z-score [35] method:

(2) $$X^{*}=\frac{X-\mu }{\sigma }$$

where X represents the original observations, μ and σ respectively represent the mean and standard deviation of the training set, $X^{*}$ denotes the standardized data.

**Figure 2:**
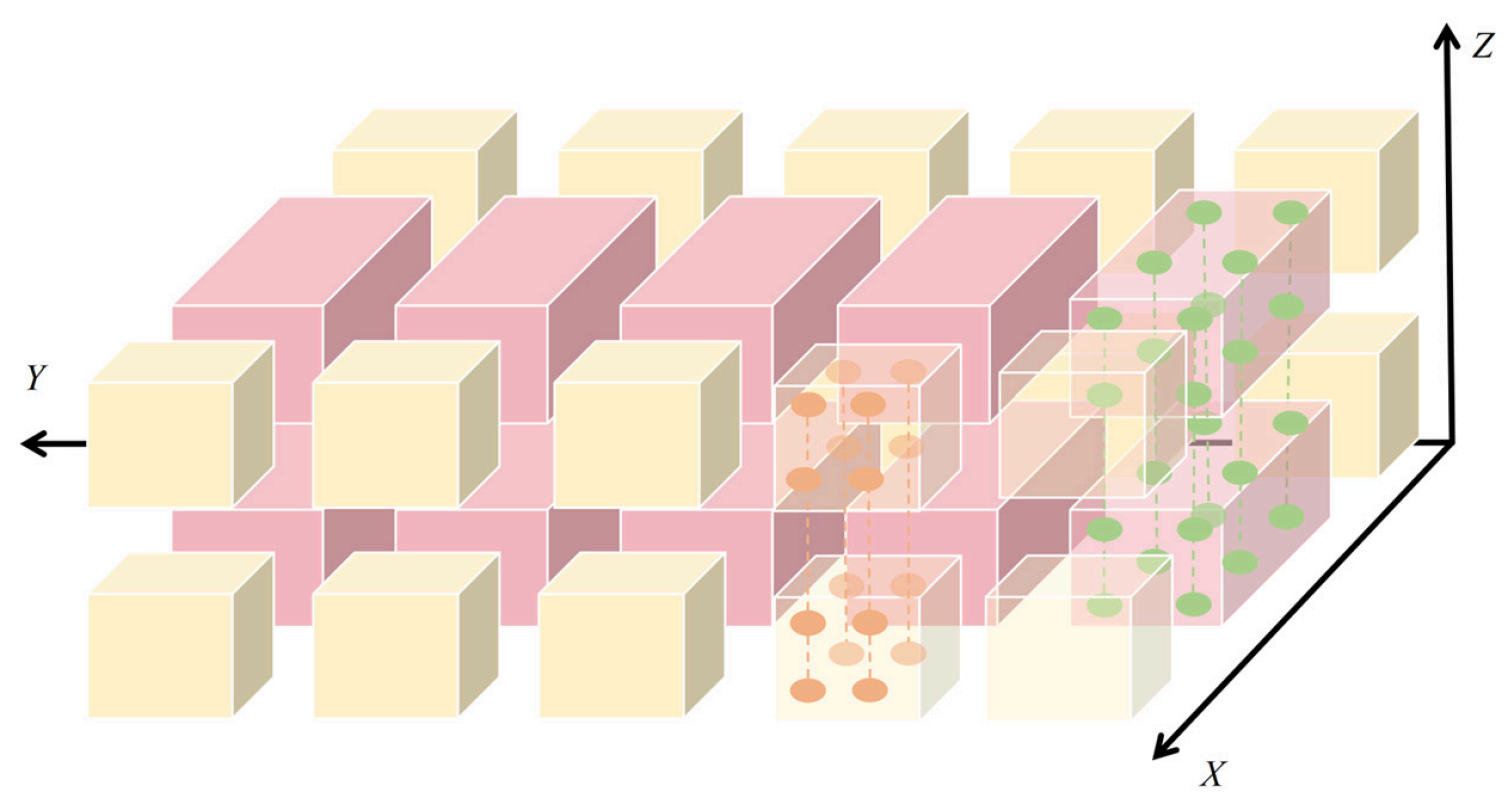
Construction of Spatial Blocks. Different colors are used to distinguish adjacent spatial blocks, while the dots indicate the temperature sensor locations within the blocks.

After preprocessing, the 280 monitoring nodes were aggregated into 30 spatial blocks according to their spatial locations, as illustrated in Figure 2. This spatial partitioning strategy was designed to transform discrete point-level measurements into regional thermal representations. The central spatial blocks were constructed using 3 × 2 × 2 monitoring nodes, whereas the edge spatial blocks were constructed using 2 × 2 × 2 monitoring nodes.

### 2.2. Spatiotemporal Correlation Analysis

**Figure 3:**
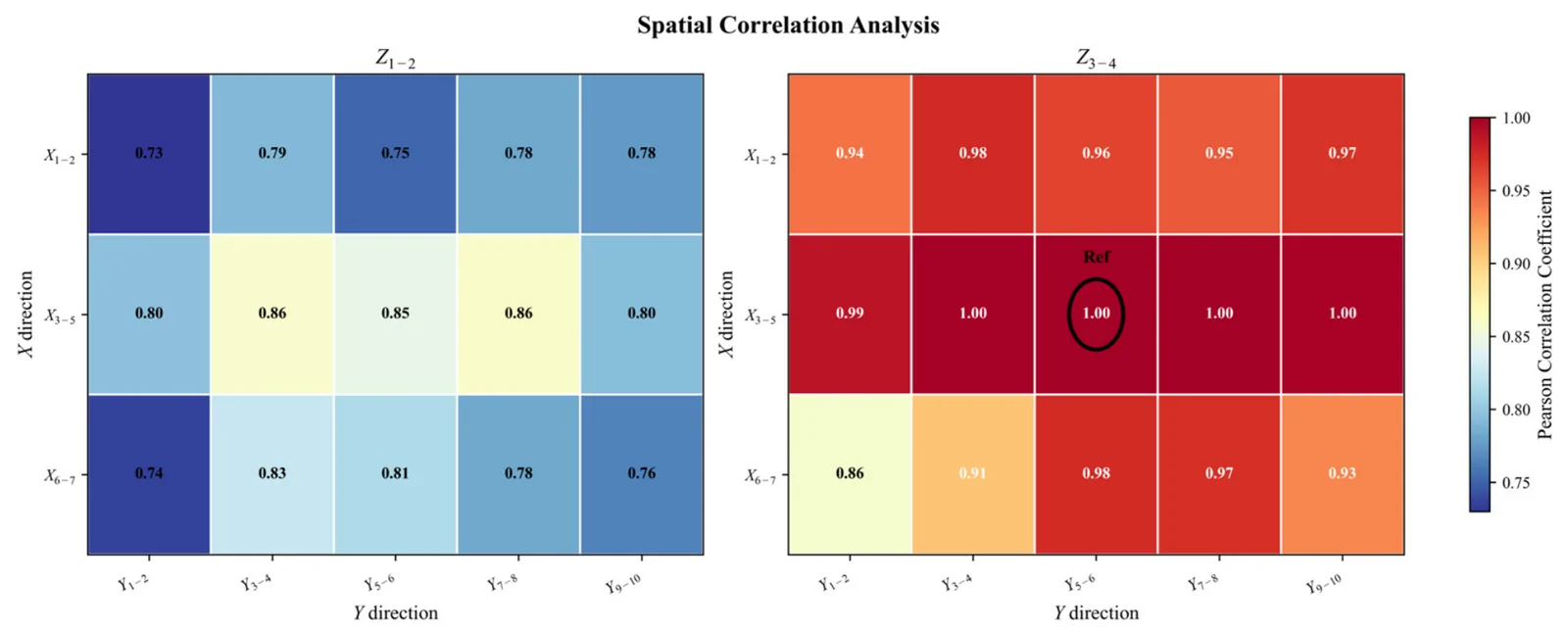
Spatial Correlation Analysis of Temperature Distribution.

The spatial dependence of thermal evolution within the grain pile was analyzed by calculating the correlations among different spatial regions. A representative block located in the central region of the grain pile was selected as the reference block, and the Pearson correlation coefficients [36] between its temperature sequence and those of other spatial blocks were calculated. As shown in Figure 3, all spatial blocks exhibited positive correlations with the reference block. The correlation coefficients of the lower-layer blocks ($Z_{1-2}$) ranged from 0.73 to 0.86, whereas those of the upper-layer blocks ($Z_{3-4}$) ranged from 0.86 to 1.00. The high correlation coefficients demonstrate that thermal evolution within the grain pile is spatially coupled rather than independent across different locations. This spatial coupling suggests that temperature changes in neighboring regions contain valuable information for characterizing regional thermal evolution and highlights the importance of incorporating spatial interactions into thermal state field forecasting.

**Figure 4:**
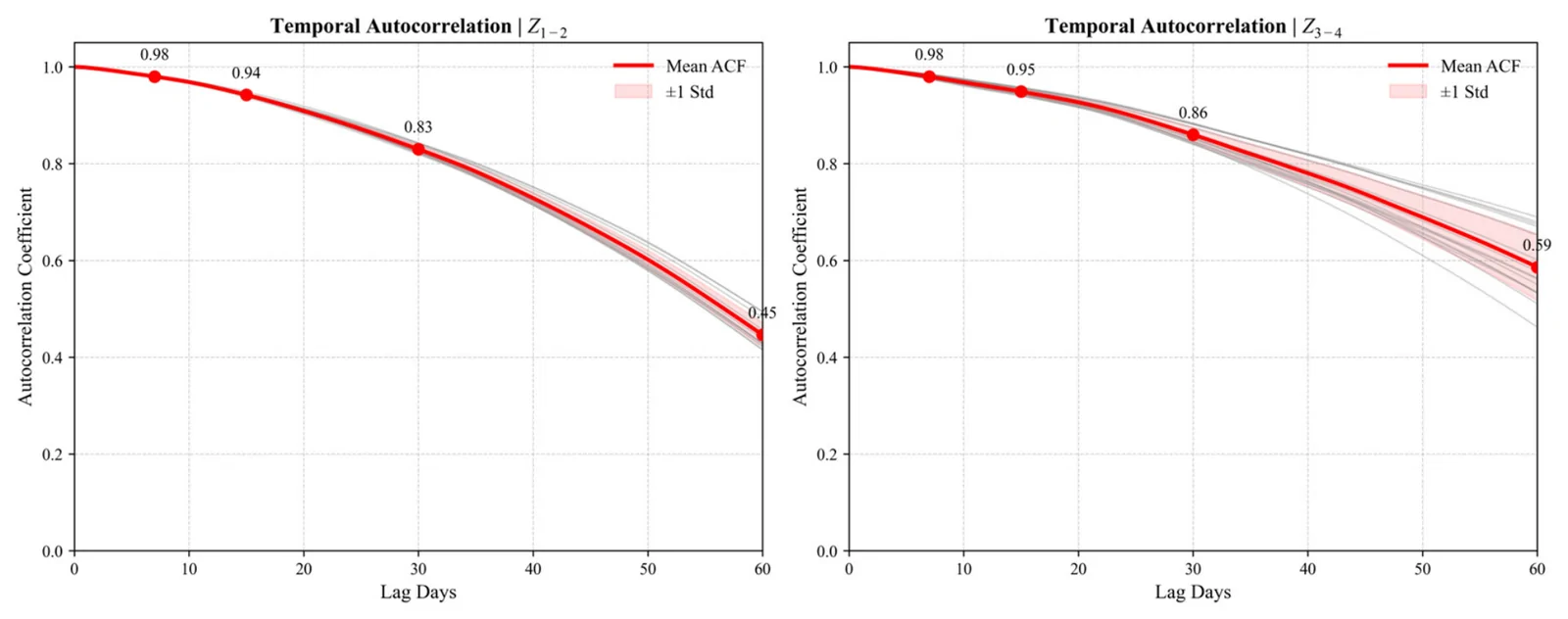
Temporal Autocorrelation Analysis of Temperature Sequences.

In addition to spatial correlations, the temporal persistence of thermal evolution was evaluated using autocorrelation analysis [37] of temperature sequences from all spatial blocks. As shown in Figure 4, the autocorrelation coefficients of both lower-layer blocks ($Z_{1-2}$) and upper-layer blocks ($Z_{3-4}$) gradually decreased with increasing lag time, while remaining relatively high over extended periods. At a 30-day lag, the average autocorrelation coefficients of the two spatial groups remained at 0.83 and 0.86, respectively. Even at a 60-day lag, the autocorrelation coefficients remained above 0.45. The observed temporal persistence indicates that the thermal-state sequences retain substantial temporal dependence over relatively long-time lags. Therefore, forecasting models should be capable of capturing long-range temporal dependencies rather than relying solely on short-term variations.

### 2.3. Thermal State Feature Set of Grain Piles

Unlike conventional analyses based on individual sensor measurements, this study takes the spatial blocks constructed above as the basic units of analysis and develops a set of thermal state features to characterize regional thermal conditions. Specifically, three categories of thermal characteristics are considered, including average temperature, temperature variance, and three-dimensional temperature gradients. These features are further integrated to establish the thermal state field, which describes the overall thermal environment of the granary in three-dimensional space. The definitions of these thermal state features are given as follows:

1. Average Temperature

The average temperature reflects the overall thermal level of a spatial region. Suppose the *i*-th spatial block contains $N_{i}$ temperature monitoring points, with corresponding temperature values $T_{i,1},T_{i,2},T_{i,3},\ldots ,T_{i,N_{i}}$. The average temperature of the spatial block is defined as:

(3) $${\bar{T}}_{i}=\frac{1}{N_{i}}\sum_{j=1}^{N_{i}}T_{i,j}$$

where ${\bar{T}}_{i}$ represents the average temperature of the *i*-th spatial block.

2. Temperature Variance

To characterize temperature dispersion within a spatial block, the temperature variance is defined as:

(4) $$V_{i}=\frac{1}{N_{i}}{\sum_{j=1}^{N_{i}}\left(T_{i,j}-{\bar{T}}_{i}\right)}^{2}$$

where $V_{i}$ represents the temperature variance of the *i*-th spatial block. A larger variance indicates greater temperature differences within the region, whereas a smaller variance indicates a more uniform thermal distribution.

3. Temperature Gradient

Heat transfer within a grain pile is closely related to spatial temperature differences [38,39]. Therefore, temperature-gradient features are introduced to characterize the directional variation and spatial non-uniformity of temperature within each block. Taking into account the three-dimensional spatial structure of the granary, the temperature gradients are calculated separately in the row, column, and layer directions. Specifically, the row direction (X) corresponds to the width of the granary, the column direction (Y) corresponds to the length of the granary, and the layer direction (Z) corresponds to the height of the grain pile. For the *i*-th spatial block unit,

(1) The row-direction temperature gradient is defined as:

(5) $$G_{X,i}=\frac{1}{M_{X}}\sum_{k=1}^{M_{X}}\left(T_{k}^{\mathrm{front}}-T_{k}^{\mathrm{back}}\right)$$

(2) The column-direction temperature gradient is defined as:

(6) $$G_{Y,i}=\frac{1}{M_{Y}}\sum_{k=1}^{M_{Y}}\left(T_{k}^{\mathrm{right}}-T_{k}^{\mathrm{left}}\right)$$

(3) The layer-direction temperature gradient is defined as:

(7) $$G_{Z,i}=\frac{1}{M_{Z}}\sum_{k=1}^{M_{Z}}\left(T_{k}^{\mathrm{upper}}-T_{k}^{\mathrm{lower}}\right)$$

where $G_{X,i}$, $G_{Y,i}$ and $G_{Z,i}$ represent the temperature gradients of the *i*-th spatial block in the row, column, and layer directions, respectively; $M_{X}$, $M_{Y}$ and $M_{Z}$ represent the number of valid adjacent temperature pairs in the row, column and layer directions, and *k* denotes the index of each adjacent temperature pair. $T_{k}^{\mathrm{front}}$ and $T_{k}^{\mathrm{back}}$ represent the temperatures of the front and back adjacent monitoring points in the row direction. $T_{k}^{\mathrm{right}}$ and $T_{k}^{\mathrm{left}}$ represent the temperatures of the right and left adjacent monitoring points in the column direction. $T_{k}^{\mathrm{upper}}$ and $T_{k}^{\mathrm{lower}}$ represent the temperatures of the upper and lower adjacent monitoring points in the layer direction.

Based on the above feature extraction process, the thermal state feature vector of the spatial block at time step *t* is defined as:

(8) $$T_{i}(t)=\{{\bar{T}}_{i}(t),V_{i}(t),G_{X,i}(t),G_{Y,i}(t),G_{Z,i}(t)\}$$

where $T_{i}(t)$ represents the thermal state features of a spatial block, including the average temperature, temperature variance, and three-dimensional temperature gradients. The thermal state features of all 30 spatial blocks are constructed following the same procedure.

## 3. Thermal State Field Forecasting Framework

**Figure 5:**
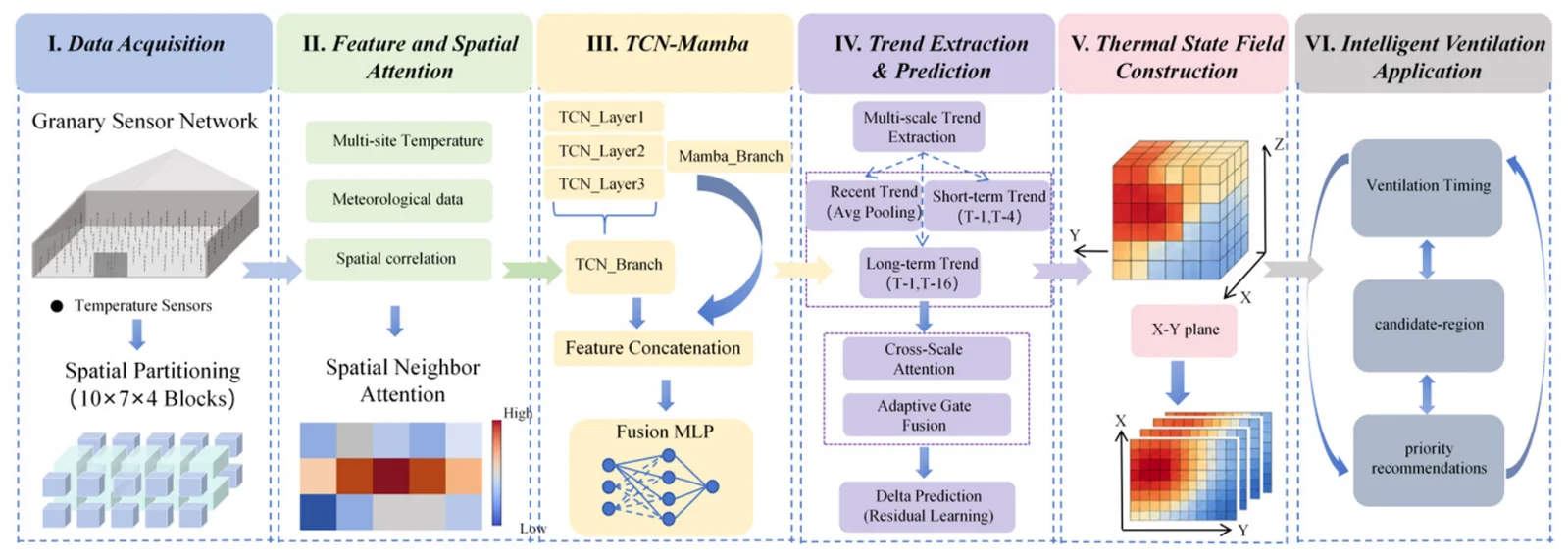
Overall Workflow of the Proposed Framework.

The overall architecture of the thermal state field forecasting framework is illustrated in Figure 5. The framework establishes a complete pipeline from multi-site sensing data acquisition to forecast-driven ventilation decision support. Specifically, the collected sensor data are first transformed into spatial thermal representations, followed by spatial feature extraction, hierarchical TCN-Mamba-based temporal modeling, Cross-Scale Trend Attention, and thermal state field reconstruction. The predicted thermal information is subsequently used to support the generation of ventilation timing, candidate-region, and priority recommendations. Detailed descriptions of each component are provided in Section 3.1, Section 3.2, Section 3.3, Section 3.4, Section 3.5 and Section 3.6.

### 3.1. Spatial Neighborhood Attention Mechanism

**Figure 6:**
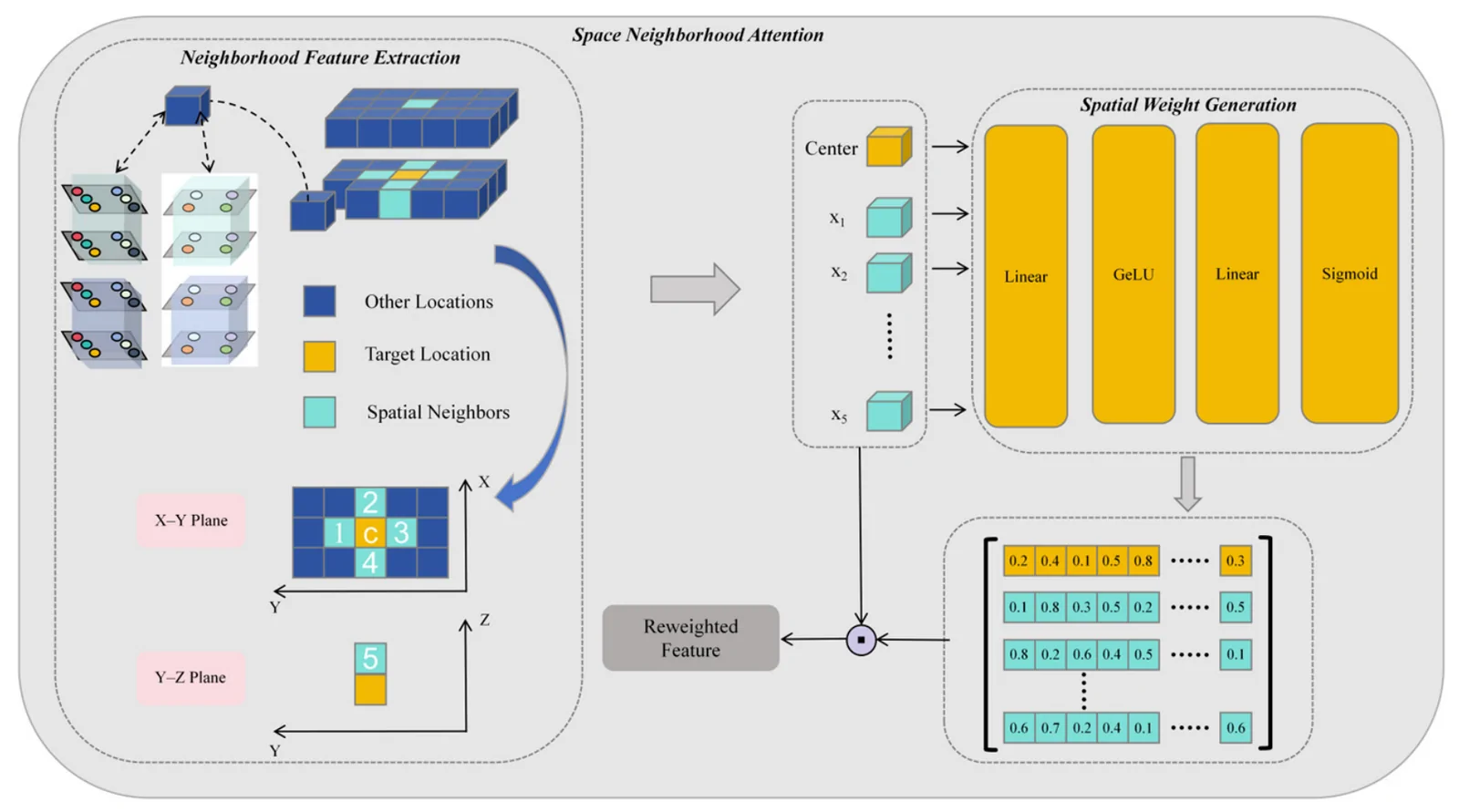
Architecture of the Spatial Neighborhood Attention Module.

As discussed in Section 2.2, temperature evolution within the grain pile exhibits clear spatial correlations, particularly among neighboring spatial blocks. To capture these local spatial dependencies before temporal modeling, a Spatial Neighborhood Attention (SNA) mechanism is introduced to generate adaptive spatial weights from neighboring-block information [40] and recalibrate the corresponding input features. Figure 6 illustrates the architecture of the SNA module, which consists of two main components: neighborhood feature extraction and spatial weight generation. For the target spatial block $B_{c}$, the target block and its valid adjacent blocks are combined to construct a local spatial neighborhood set:

(9) $$N=\{B_{c},B_{1},B_{2},\ldots ,B_{k}\},\;k\leq 5$$

where *N* denotes the neighborhood set, and k denotes the number of valid neighboring blocks. For boundary blocks, the number of neighboring blocks may be smaller than that of interior blocks, and the subsequent spatial weights are calculated only over the valid neighboring blocks.

For each neighborhood block, first extract its corresponding feature vector and form a neighborhood feature set:

(10) $$X_{f}=\{x_{c},x_{1},\ldots ,x_{k}\}\in R^{(k+1)\times C}$$

where $x_{c}$ is the target block feature, $x_{k}$ is the valid neighborhood block feature, *C* represents the feature dimension.

Due to the limited ability of traditional spatial modeling methods with fixed neighborhood relationships to capture sample-varying heterogeneous spatial correlations, a spatial weight matrix is generated through shared multi-layer perceptrons:

(11) $$W=\sigma (W_{2}(\mathrm{GeLU}(W_{1}X_{f}+b_{1}))+b_{2})$$

where $X_{f}$ denotes input neighborhood features; $W_{1}$, $b_{1}$ and $W_{2}$, $b_{2}$ are linear layer parameters. GeLU is selected rather than ReLU due to its smooth gradient curve, which avoids dead neurons and better fits subtle continuous spatial dependencies between blocks. σ normalizes weights to [0, 1]. After obtaining the spatial weights, element-by-element weighting is applied to the original neighborhood features:

(12) $$S=W⊙X_{f}$$

where ⊙ denotes the element-wise multiplication operation. Through this operation, the model can automatically enhance neighborhood information highly correlated with the thermal state of the target area, while suppressing interference from noise neighborhoods or anomalous areas. Additionally, S serves as input for the subsequent Hierarchical TCN and Mamba dual-branch networks, enabling joint modeling of spatial correlation and time dependence.

### 3.2. Hierarchical TCN-Mamba Encoder

**Figure 7:**
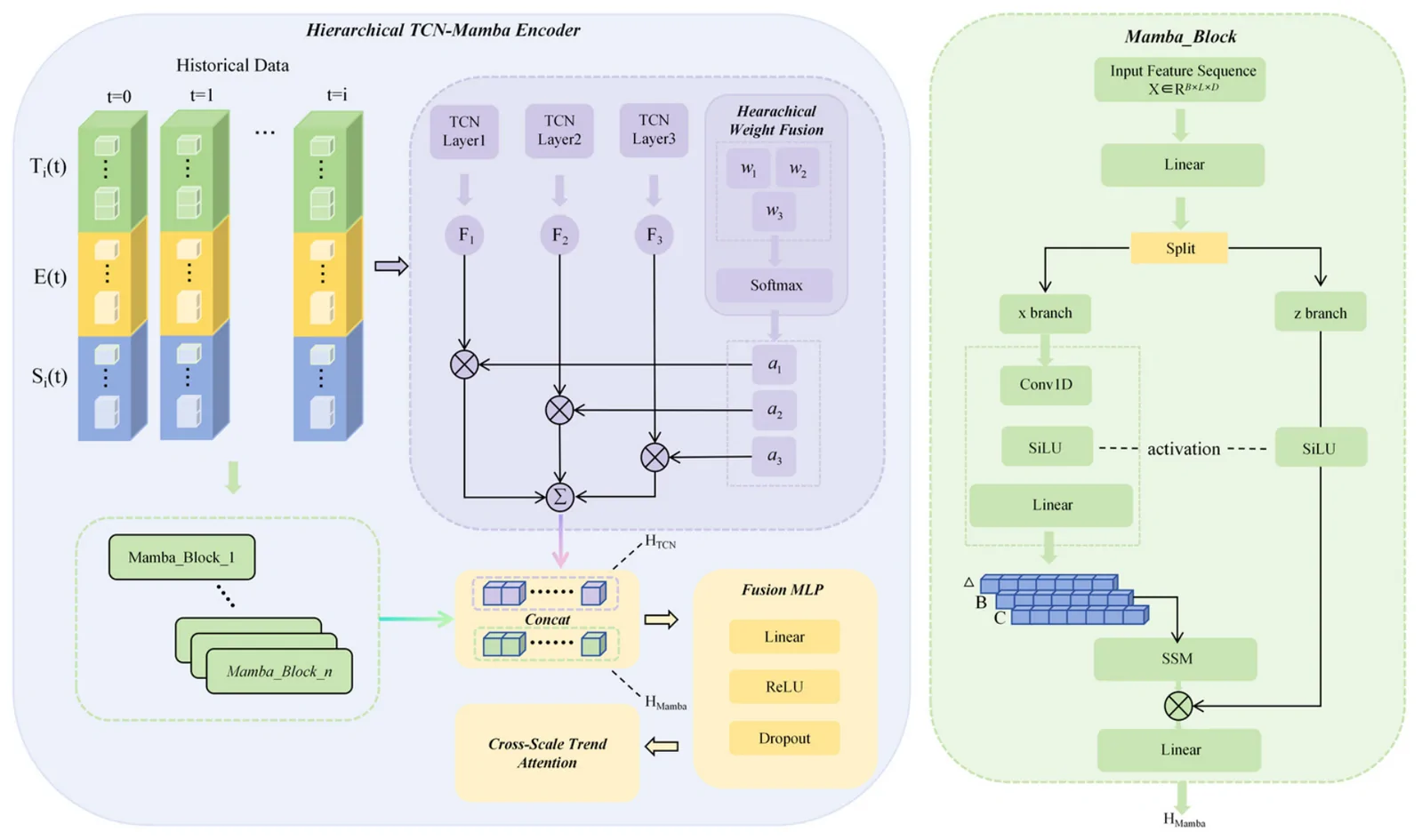
Architecture of the Hierarchical TCN-Mamba Framework.

The thermal evolution of grain piles exhibits both short-term local fluctuations and long-term variation patterns. Therefore, a Hierarchical TCN–Mamba Encoder is developed to extract temporal features at different time scales. Figure 7 presents the overall architecture and computational workflow of the proposed encoder. The encoder consists of four main stages: input sequence construction, hierarchical multi-scale feature extraction, long-term state evolution modeling, and dual-branch feature fusion. First, the thermal state features, meteorological, and spatial correlation features are organized into a unified input sequence. The TCN branch extracts multi-scale local temporal patterns through hierarchical dilated convolutions [41], while the Mamba branch captures long-range temporal dependencies through selective state-space modeling. Finally, the complementary representations generated by the two branches are fused to construct a unified temporal representation, which is subsequently fed into the Cross-Scale Trend Attention module.

The Hierarchical TCN–Mamba encoder first constructs a unified historical input sequence. The model input consists of three types of information: the thermal-state features of the target spatial block, meteorological variables, and spatial features generated by the SNA module, denoted as $T_{i}(t),E(t),S_{i}(t)$, respectively. The meteorological variable vector is defined as:

(13) $$E(t)=[T_{\mathrm{out}}(t),H_{\mathrm{out}}(t)]$$

where $T_{\mathrm{out}}(t)$ and $H_{\mathrm{out}}(t)$ represent the outdoor air temperature and humidity, respectively. The input sequence is represented as follows:

(14) $$X_{\mathrm{in}}=\{[T_{i}(t-L+1),E(t-L+1),S_{i}(t-L+1)],\ldots ,[T_{i}(t),E(t),S_{i}(t)]\}$$

where $X_{\mathrm{in}}$ denotes the input sequence, *t* is the current time step, and *L* is the length of the historical input window.

The Hierarchical TCN module then employs a three-layer dilated convolution structure to capture temporal variation patterns at different scales. As the dilation rate increases layer by layer, the receptive field is progressively expanded, allowing the network to incorporate information over broader temporal ranges. However, relying solely on the output of the final convolutional layer may lead to the loss of complementary temporal information extracted at intermediate scales. Therefore, the proposed hierarchical TCN preserves the feature representations generated by all convolutional layers and introduces learnable fusion coefficients to adaptively integrate multi-scale temporal information. Specifically, the learnable parameters $w_{1},w_{2},w_{3}$ are normalized by the Softmax function to generate the fusion coefficients:

(15) $$a_{i}=\frac{\exp(w_{i})}{\sum_{j=1}^{3}\exp(w_{j})}$$

where $a_{i}$ denotes the normalized fusion coefficient assigned to the *i*-th TCN layer. The normalized fusion coefficients are subsequently applied to aggregate the feature representations extracted at different temporal scales, and the output of the hierarchical TCN branch is formulated as:

(16) $$H_{\mathrm{TCN}}=\sum_{i=1}^{3}F_{i}a_{i}$$

where $F_{i}$ represents the temporal feature representation generated by the *i*-th TCN layer, $H_{\mathrm{TCN}}$ represents the feature representations extracted by the Hierarchical TCN branch. The adaptive weighted aggregation preserves complementary temporal information extracted at different scales, thereby enhancing the multi-scale temporal representation capability of the encoder.

The Mamba block performs feature projection, branch splitting, selective state-space modeling, and gated feature generation in a sequential manner. Given the input sequence $X_{\mathrm{in}}$, the features are first projected into a latent space through a linear transformation and subsequently divided into a state modeling branch and a gating branch. The projected features are expressed as:

(17) $$\left[X_{s},X_{g}]=\mathrm{Split}(W_{\mathrm{in}}X_{\mathrm{in}}\right)$$

where $W_{\mathrm{in}}$ represents the input projection matrix. The projected features are divided into two branches: $X_{s}$ is the state modeling branch, and $X_{g}$ is the gating branch.

In the $X_{s}$ branch, depthwise causal convolution is first applied to extract local continuous temporal patterns while preserving temporal causality. The extracted features are then processed by the SiLU activation function and a linear projection to generate the input-dependent state-space parameters:

(18) $$\Delta ,B,C=W_{p}(\mathrm{SiLU}(\mathrm{Conv}(X_{s})))+b_{p}$$

where Δ denotes the dynamic time-step parameter, *B* represents the input-to-state mapping parameter, and *C* denotes the state-to-output mapping parameter. Unlike conventional state-space models with fixed parameters, Δ, *B* and *C* are dynamically generated from the current input, enabling adaptive state transitions according to different temporal patterns. The generated parameters are then incorporated into the selective state-space model for recursive state updating. At time step *t*, the hidden state is updated as follows:

(19) $$h_{t}=A_{t}h_{t-1}+B_{t}x_{t}$$

where $h_{t}$ denotes the current hidden state, $A_{t}$ is the state transition matrix, $B_{t}$ represents the input mapping matrix, and $x_{t}$ stands for the input feature vector at the current moment. Through the recursive state update mechanism, the model continuously integrates historical state information with the current input, thereby establishing long-range temporal dependencies. The corresponding state output is computed as:

(20) $$y_{t}=C_{t}h_{t}+Dx_{t}$$

where $C_{t}$ denotes the state-to-output mapping matrix, and *D* represents the skip connection term for retaining direct information contained in the current input.

Meanwhile, the gating branch $X_{g}$ generates adaptive gating features through the SiLU activation function. The gating features are multiplied element-wise with the SSM output, and the fused features are projected back to the original feature dimension, yielding the output of the Mamba branch:

(21) $$H_{\mathrm{Mamba}}=W_{o}(\mathrm{SiLU}(X_{z})⊗y_{t})$$

where $W_{o}$ denotes the output projection matrix and $H_{\mathrm{Mamba}}$ represents the feature representation extracted by the Mamba branch.

To integrate the complementary representations generated by the two branches, their outputs are concatenated and mapped through a fusion MLP to construct a unified temporal representation:

(22) $$H_{f}=\mathrm{Dropout}(\mathrm{ReLU}(W_{f}(H_{\mathrm{TCN}}⊕H_{\mathrm{Mamba}})+b_{f}))$$

where $H_{f}$ represents the fused feature representation, $W_{f}$ and $b_{f}$ denote the weight matrix and bias term of the fusion MLP, respectively.

Finally, the fused feature $H_{f}$ is fed into the cross-scale trend attention module to capture the relationships among recent, short and long-term temporal patterns.

### 3.3. Cross-Scale Trend Attention

**Figure 8:**
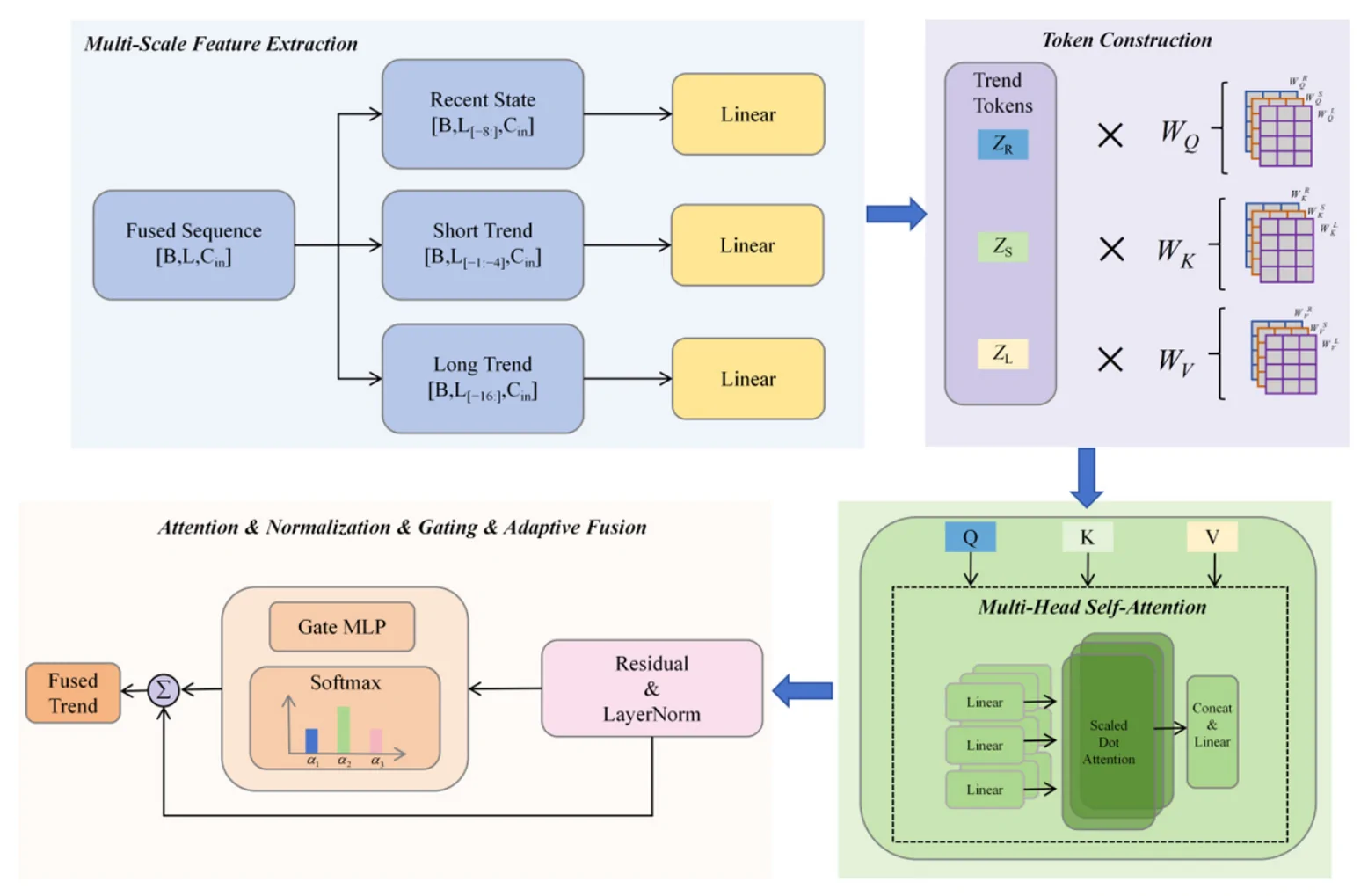
Architecture of the Cross-Scale Trend Attention Module.

The Cross-Scale Trend Attention module is organized into four sequential stages, as illustrated in Figure 8, and is designed to capture dependencies among temporal trends at different scales. The fused sequence is first decomposed into recent, short and long-term trend representations in the Multi-Scale Feature Extraction stage. These representations are then projected into a unified embedding space, where trend tokens are constructed during the Token Construction stage. Subsequently, the constructed tokens interact through Multi-Head Self-Attention, enabling the extraction of cross-scale dependencies. Finally, the attended features are refined by Residual & LayerNorm and adaptively integrated through the Gating & Adaptive Fusion stage, yielding a unified temporal representation for subsequent prediction.

In the Multi-Scale Feature Extraction stage shown in Figure 8, the fused sequence is first decomposed into recent, short and long-term trend representations. To reduce the feature distribution differences across different temporal scales, independent linear projections are employed to map them into a unified embedding space:

(23) $$\left\{\begin{array}{l} Z_{R}=W_{R}R+b_{R}\\ Z_{S}=W_{S}R+b_{S}\\ Z_{L}=W_{L}R+b_{L} \end{array}\right.$$

The projected representations are subsequently combined to construct the trend token sequence:

(24) $$Z_{\mathrm{trend}}=[Z_{R},Z_{S},Z_{L}]\in R^{B\times 3\times d}$$

where $Z_{\mathrm{trend}}$ represents the multi-scale trend embedding, $Z_{R}$, $Z_{S}$ and $Z_{L}$ denote the recent, short and long-term trend representations, respectively, *B* denotes the batch size and *d* denotes the trend embedding dimension.

The constructed trend tokens are subsequently fed into the Multi-Head Self-Attention stage shown in Figure 8. Linear transformations are first performed to generate the query, key, and value matrices:

(25) $$\left\{\begin{array}{l} Q=Z_{\mathrm{trend}}W_{Q}\\ K=Z_{\mathrm{trend}}W_{K}\\ V=Z_{\mathrm{trend}}W_{V} \end{array}\right.$$

The generated *Q*, *K* and *V* are then processed by scaled dot-product attention to model the dependencies among different temporal trends:

(26) $$H_{R,S,L}=S\text{oftmax}(\frac{QK^{T}}{\sqrt{d}})V$$

To preserve the original trend information and improve training stability, residual connections and layer normalization are introduced. Since the relative importance of different temporal scales may vary during thermal evolution, a gating fusion network is further used to generate adaptive weights for the three trend representations:

(27) $$[g_{R},g_{S},g_{L}]=S{\mathrm{oftmax}(\mathrm{MLP}(\mathrm{LayerNorm}(H}_{R,S,L}+Z_{\mathrm{trend}})))$$

where $g_{R}$, $g_{S}$ and $g_{L}$ denote the adaptive fusion weights for the recent, short and long-term trend representations, respectively. Multi-scale trend fusion is then performed using the derived weights:

(28) $$H_{\mathrm{trend}}=g_{R}h_{R}+g_{S}h_{S}+g_{L}h_{L}$$

where $H_{\mathrm{trend}}$ represents the fused trend representation, and $h_{R}$, $h_{S}$, $h_{L}$ denote the feature representations of the recent, short and long-term trends, respectively.

### 3.4. Residual Incremental Prediction

Thermal states in grain piles evolve gradually and exhibit strong temporal continuity, with future states generally remaining close to the current state. To enhance the stability of long-term forecasting, the model adopts a residual incremental learning strategy that predicts changes relative to the current thermal state rather than directly forecasting absolute future values, which is consistent with the gradual evolution characteristics of grain thermal states.

Specifically, the prediction head is first applied to map the fused trend representation to the predicted thermal-state increment:

(29) $$\hat{Y}=Y_{\mathrm{last}}+f_{\mathrm{head}}(H_{\mathrm{trend}})⊙\sigma_{X}$$

where $f_{\mathrm{head}}(H_{\mathrm{trend}})$ denotes the predicted increment, $Y_{\mathrm{last}}$ represents the thermal state at the last time step of the historical window, $\sigma_{X}$ represents the scale recovery parameter corresponding to the input sequence, and ⊙ denotes element-wise multiplication.

Accordingly, the corresponding prediction target is defined as:

(30) $$\Delta Y=Y_{\mathrm{future}}-Y_{\mathrm{last}}$$

which represents the change in the future thermal state relative to the current state.

### 3.5. Construction of Thermal State Field

The proposed model predicts the average temperature, temperature variance, and three-dimensional temperature gradients for each spatial block at future time steps. Since these thermal-state variables are obtained at the discrete spatial-block level, spatial interpolation is introduced to construct continuous field representations for visualization within the grain pile. The interpolation process is only used for visual presentation and does not participate in model training, quantitative evaluation, or ventilation decision-making. The predicted thermal variables remain the original outputs of the forecasting model.

Let the average temperature and temperature variance of all spatial blocks at future time steps be:

(31) $$\left\{\begin{array}{l} {\bar{T}}_{i}=\{{\bar{T}}_{1},{\bar{T}}_{2},\ldots ,{\bar{T}}_{N}\}\\ V_{i}=\{V_{1},V_{2},\ldots ,V_{N}\} \end{array}\right.$$

where *N* denotes the total number of spatial blocks inside the granary. According to the spatial coordinates of the 30 blocks, the predicted block-level thermal variables are first mapped back to the 3 × 5 × 2 spatial structure of the grain bulk, with each Z layer corresponding to a discrete 3 × 5 block grid in the X−Y plane. To obtain continuous spatial representations, third-order spline interpolation is independently applied within the X−Y plane of each Z layer, using the predicted values of the spatial blocks as discrete interpolation nodes. Specifically, piecewise cubic polynomials are used to interpolate the spatial variation between adjacent nodes, while the interpolated values and their first- and second-order derivatives remain continuous at the connections between adjacent interpolation intervals. Since only two block groups are available along the Z direction, the two layers are processed independently without cross-layer interpolation. The average temperature and temperature variance fields are thereby obtained as:

(32) $$\left\{\begin{array}{l} T_{z}(x,y)=L_{xy}^{\left(3\right)}({\bar{T}}_{z})\\ V_{z}(x,y)=L_{xy}^{\left(3\right)}(V_{z}) \end{array}\right.,z=1,2$$

where $L_{xy}^{\left(3\right)}(•)$ denotes the third-order spline interpolation operator applied within the X−Y plane, and *z* = 1, 2 denotes the two spatial layers.

**Figure 9:**
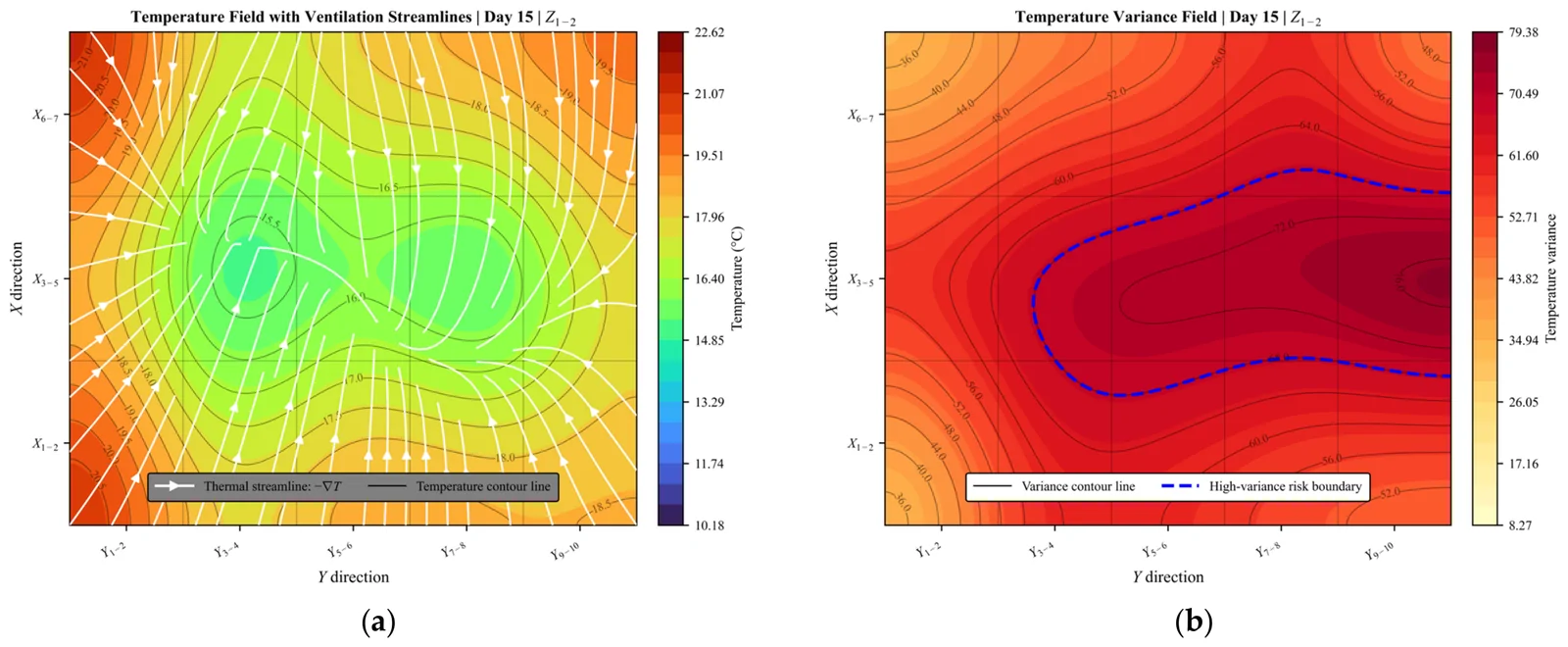
Thermal State Field Visualization for Ventilation Decision-Making: (**a**) presents the mean temperature field, where the white streamlines indicate the estimated potential heat-transfer directions. (**b**) illustrates the distribution of the temperature variance field, where the blue dashed contour highlights regions with relatively high temperature variance.

The reconstructed average temperature field characterizes the spatial distribution of thermal levels, while the temperature variance field reflects spatial thermal heterogeneity. The predicted three-dimensional temperature gradients further characterize directional thermal variations within each spatial block. According to Fourier’s law of heat conduction, the heat flux is directed opposite to the temperature gradient:

(33) $$\left\{\begin{array}{l} q=-k\nabla T\\ G=[G_{X},G_{Y},G_{Z}] \end{array}\right.$$

where *q* denotes the heat flux density vector, *k* denotes the thermal conductivity, ∇*T* denotes the three-dimensional temperature gradient, and *G* represents the block-level temperature-gradient vector predicted by the model. Since these gradient predictions are defined at discrete spatial blocks, spatial interpolation is applied only to convert the discrete block-level gradients into a continuous field representation for visualization. The original gradient predictions are retained unchanged for quantitative evaluation and VDI calculation. For layer-wise visualization, the predicted $G_{X}$ and $G_{Y}$ are interpolated within each X–Y plane, and their negative direction is used to represent the potential heat-transfer direction field:

(34) $$D_{z}(x,y)=-[L_{xy}^{\left(3\right)}(G_{X}),L_{xy}^{\left(3\right)}(G_{Y})],\;z=1,2$$

where $D_{z}(x,y)$ denotes the estimated potential heat-transfer direction field in the X–Y plane of layer z, and $L_{xy}^{\left(3\right)}(G_{X})$ and $L_{xy}^{\left(3\right)}(G_{Y})$ denote the interpolated X and Y-direction gradient components, respectively. Figure 9 jointly presents the average temperature field, potential heat-transfer directions, and temperature variance field.

### 3.6. Event-Triggered Ventilation Decision-Support Framework

The event-triggered ventilation component is formulated as an illustrative forecast-driven decision-support framework based on the predicted thermal state field. The predicted average temperature, temperature variance, and three-dimensional temperature gradients are integrated to characterize future thermal evolution and quantify the relative ventilation demand across different spatial regions. Based on the calculated Ventilation Demand Index (VDI), predefined triggering conditions, and priority thresholds, the framework identifies regions with potential ventilation demand and provides corresponding region-level ventilation priority recommendations.

First, a Ventilation Demand Index (VDI) is constructed to characterize the relative ventilation demand:

(35) $$VDI_{i}^{t}=w_{t}R_{T,i}^{t}+w_{r}R_{\Delta T,i}^{t}+w_{v}R_{\Delta V,i}^{t}+w_{g}R_{G,i}^{t}$$

where $R_{T,i}^{t},R_{\Delta T,i}^{t},R_{\Delta V,i}^{t},R_{G,i}^{t}$ denote the Min–Max normalized predicted average temperature, positive temperature trend, positive variance trend, and temperature-gradient magnitude, respectively. The weighting coefficients $w_{t}$, $w_{r}$, $w_{v}$, and $w_{g}$ correspond to the average temperature, temperature trend, variance trend, and temperature-gradient magnitude, respectively. The temporal trend terms, temperature-gradient magnitude and Min–Max normalization are calculated as follows:

(36) $$R_{T,i}^{t}=\frac{{\bar{T}}_{i}^{t}-{\bar{T}}_{\min}}{{\bar{T}}_{\max}-{\bar{T}}_{\min}}$$

(37) $$R_{\Delta T,i}^{t}=N[\max(\frac{{\bar{T}}_{i}^{t}-{\bar{T}}_{i}^{t-L}}{L},0)]$$

(38) $$R_{\Delta V,i}^{t}=N[\max(\frac{V_{i}^{t}-V_{i}^{t-L}}{L},0)]$$

(39) $$R_{G,i}^{t}=N[\sqrt{{\left(G_{x,i}^{t}\right)}^{2}+{\left(G_{y,i}^{t}\right)}^{2}+{\left(G_{z,i}^{t}\right)}^{2}}]$$

(40) $$N(x)=\frac{x-x_{\min}}{x_{\max}-x_{\min}}$$

where *L* denotes the temporal trend calculation window. Since the thermal state of the grain bulk evolves gradually due to thermal inertia, the temporal trend is calculated over an *L*-day interval rather than between two consecutive days, thereby reducing the influence of short-term fluctuations and providing a more stable estimation of the long-term thermal evolution and N(•) denotes Min–Max normalization, where $x_{\min}$ and $x_{\max}$ represent the minimum and maximum values of the corresponding VDI component, respectively. For the illustrative decision-support analysis, the training-set ranges of [8, 25], [0, 0.30], [0, 3.50], and [5, 20] are used for the predicted average temperature, positive temperature trend, positive variance trend, and temperature-gradient magnitude, respectively. These ranges provide a consistent dimensionless scale for weighted VDI aggregation and remain unchanged throughout the 30-day decision-support analysis.

A larger VDI value represents a higher relative ventilation demand under the proposed indicator formulation and therefore corresponds to a higher recommended ventilation priority. The VDI is then combined with thermal-evolution indicators to define an initial triggering condition for identifying candidate regions with potential ventilation demand. Specifically, a binary triggering indicator $I_{i}^{t}$ is introduced to determine whether spatial region *i* simultaneously satisfies the predefined temperature-growth, *L*-day temperature-rise, and VDI conditions at time step *t*:

(41) $$I_{i}^{t}=\left\{\begin{array}{l} 1,(\frac{{\bar{T}}_{i}^{t}-{\bar{T}}_{i}^{t-L}}{L}>\theta_{T})\wedge ({\bar{T}}_{i}^{t}-{\bar{T}}_{i}^{t-L}>\theta_{C})\wedge (VDI_{i}^{t}>\theta_{V})\\ 0,\;otherwise \end{array}\right.$$

where $I_{i}^{t}$ = 1 indicates that the three predefined conditions are simultaneously satisfied, $I_{i}^{t}$ = 0 indicates that at least one condition is not satisfied. $\theta_{T}$, $\theta_{C}$ and $\theta_{V}$ denote the threshold values of the average temperature growth rate, *L*-day temperature-rise, and VDI, respectively. The average temperature growth rate reflects the warming tendency of a spatial region over the temporal window *L*, while the *L*-day temperature rise characterizes the net temperature increase over the same period.

Furthermore, satisfying the triggering conditions at a single time step may result from short-term fluctuations. Therefore, a continuous triggering strategy is introduced to improve the temporal consistency of the generated recommendations. A ventilation recommendation is activated only when the above conditions are continuously satisfied within a consecutive time window:

(42) $$E_{i}^{t}=\left\{\begin{array}{l} 1,\sum_{k=t-n+1}^{t}I_{i}^{k}=n\\ 0,\;otherwise \end{array}\right.$$

where *n* denotes the length of the continuous triggering window. When $E_{i}^{t}=1$, the corresponding region is retained as a candidate ventilation region and assigned a recommended priority level according to the VDI. The priority assignment is defined as:

(43) $$P_{i}^{t}=\left\{\begin{array}{l} P_{low},\;VDI_{i}^{t}\leq \theta_{M}\\ P_{medium},\;\theta_{M}<VDI_{i}^{t}\leq \theta_{H}\\ P_{high},\;VDI_{i}^{t}>\theta_{H} \end{array}\right.$$

where $P_{low}$, $P_{medium}$ and $P_{high}$ denote the recommended low, medium, and high ventilation-priority levels, respectively, while $\theta_{M}$ and $\theta_{H}$ denote the corresponding decision-priority thresholds.

The parameter settings of the event-triggered ventilation decision-support framework are summarized in Table 2.

The baseline VDI weights were set as $w_{t}$ = 0.15, $w_{r}$ = 0.35, $w_{v}$ = 0.25, $w_{g}$ = 0.25, subject to $w_{t}+w_{r}+w_{v}+w_{g}=1$. Owing to the absence of field ventilation-response data, these weights were specified as an engineering baseline rather than an experimentally optimized solution. The temperature-trend term was assigned the largest weight because it directly characterizes the warming tendency over the forecasting horizon. The variance-trend and temperature-gradient terms were assigned equal intermediate weights to characterize the evolution of thermal nonuniformity and spatial heat migration, respectively. The temperature-level term received a smaller weight because temperature change is additionally considered through the growth-rate and *L*-day temperature-rise triggering conditions.

The triggering parameters $\theta_{V}$, $\theta_{T}$, $\theta_{C}$, and n impose minimum requirements on the integrated demand, temperature-growth rate, *L*-day temperature rise, and temporal persistence, respectively. Their baseline values were specified as engineering settings for the present dataset rather than field-calibrated optimal values.

A one-at-a-time sensitivity analysis was conducted to examine how the decision-support output responds to variations in the four VDI weights and four triggering parameters. For the weight analysis, the unit-sum constraint was maintained throughout all tested configurations. When one weight was changed from $w_{i}$ to $w_{i}^{\prime }$, the remaining weights were proportionally renormalized as:

(44) $$w_{j}^{\prime }=w_{j}\frac{1-w_{i}^{\prime }}{1-w_{i}},\;j\neq i$$

thereby ensuring that the sum of weights remained equal to 1 for every tested configuration and preventing changes in the overall VDI scale caused solely by variations in the total weight sum. Each triggering parameter was varied independently while all other triggering parameters remained at their baseline values. Sensitivity was quantified by the decision-triggering ratio, defined as the percentage of region-day samples for which an event-triggered ventilation recommendation was generated.

## 4. Experimental Results and Discussion

### 4.1. Evaluation Metrics

For the grain-pile thermal-state prediction task, RMSE and MAE are adopted as model evaluation metrics. RMSE measures the overall deviation between the predicted and ground-truth values and gives greater weight to larger prediction errors, while MAE reflects the average magnitude of the absolute prediction errors. The definitions of the evaluation metrics are given as follows:

(45) $$RMSE=\sqrt{\frac{1}{N}\sum_{i=1}^{N}{\left(y_{i}-{\hat{y}}_{i}\right)}^{2}}$$

(46) $$MAE=\frac{1}{N}\sum_{i=1}^{N}\left|y_{i}-{\hat{y}}_{i}\right|$$

where $y_{i}$ and ${\hat{y}}_{i}$ denote the ground-truth value and predicted value of the i-th sample, respectively, and *N* represents the total number of test samples. Smaller RMSE and MAE values indicate lower prediction errors.

In addition to the error metrics, statistical comparisons between the proposed model and the baseline models are conducted using a two-sided Wilcoxon signed-rank test. For each spatial block, the RMSE and MAE values obtained from three independent runs are first averaged, and the resulting values from the same 30 spatial blocks are used as paired observations. This non-parametric paired test is used to assess whether the performance differences between two models are consistently supported across the spatial blocks without assuming a normal distribution of the paired differences. Since the proposed model is compared with five baseline models for each predicted variable and evaluation metric, the resulting *p*-values are adjusted using the Holm method to account for multiple comparisons. An adjusted *p*-value below 0.05 is considered statistically significant.

### 4.2. Experimental Settings

To ensure a leakage-safe evaluation protocol, the complete dataset consisting of 732 daily observations was divided chronologically into training, validation, and test subsets with a ratio of 70%:15%:15%, corresponding to 512, 110, and 110 observations, respectively. This resulted in 432 training windows and 30 validation windows, which were generated independently within their corresponding subsets. During the training stage, an 80-day historical sequence was used to predict the next-day thermal state. During the testing stage, a rolling one-step-ahead forecasting strategy was adopted to generate consecutive predictions over a 30-day period. Specifically, the predicted block-level thermal-state features, including average temperature, temperature variance, and three-dimensional temperature gradients, were appended to the historical input sequence as the thermal-state information for the newly predicted time step, while the oldest time step was removed to maintain the fixed 80-day input length. Meanwhile, the spatial neighborhood topology remained unchanged according to the predefined spatial block partition. At each rolling step, the neighborhood feature matrix was reconstructed using the updated thermal state features of the target block and its valid neighboring blocks, and the updated neighborhood features were subsequently used to construct the input of the next forecasting step. During this process, only historical meteorological variables available within the 80-day input window were used as auxiliary information, and no future meteorological observations beyond the current prediction step were introduced into the forecasting process. Consequently, neither the historical input sequences nor the rolling forecasting process crossed the boundaries between different subsets. Moreover, the normalization parameters were estimated exclusively from the training subset and subsequently applied unchanged to the validation and test subsets.

The proposed model employed three causal dilated convolution layers in the TCN branch and four Mamba blocks in the Mamba branch. The remaining network architecture and training hyperparameters are summarized in Table 3. To maintain consistent training conditions for model comparison, all learning-based models used the same training hyperparameters, including batch size, learning rate, dropout rate, maximum number of epochs, optimizer, and early-stopping settings. All experiments were implemented in Python 3.10 using the PyTorch 2.1.1 deep learning framework and conducted on a workstation equipped with an NVIDIA GeForce RTX 4090 GPU.

### 4.3. Discussion of Prediction Results

#### 4.3.1. Model Comparison Across All 30 Spatial Blocks

Table 4 and Table 5 present the prediction results for average temperature, temperature variance, and three-dimensional temperature gradients across all 30 spatial blocks. For each independent run, RMSE and MAE were calculated separately for each spatial block and then averaged across the 30 blocks. The final results are reported as the mean ± standard deviation of three independent runs. For average temperature, the proposed model achieves the lowest RMSE and MAE of 0.2988 ± 0.0374 and 0.2591 ± 0.0330, respectively, which are 0.0251 and 0.0244 lower than those of the competitive TCN. For temperature variance, the proposed model obtains an RMSE of 0.5573 ± 0.0112 and an MAE of 0.4270 ± 0.0093; compared with LSTM, the corresponding errors are reduced by 0.0210 and 0.0222. More evident differences are observed for the three-dimensional temperature gradients. The proposed model achieves RMSE values of 0.0380 ± 0.0009, 0.0442 ± 0.0031, and 0.0788 ± 0.0050 in the X, Y, and Z directions, respectively, which are 0.0026, 0.0017, and 0.0111 lower than those of TCN.

Table 6 further presents the paired statistical comparisons across the 30 spatial blocks. After Holm correction, significant differences are observed for multiple baseline–variable combinations. For average temperature, both RMSE and MAE show significant differences against Informer and iTransformer, while for temperature variance, significant differences are found against Informer, iTransformer, and TCN. Statistical significance is more broadly observed for the three-dimensional temperature gradients, where the proposed model shows significant error reductions over most baseline models. Some comparisons with competitive baselines do not reach statistical significance, consistent with the relatively close errors reported in Table 4 and Table 5. Overall, the aggregated results across the 30 spatial blocks and the paired statistical comparisons show that the proposed model achieves relatively low prediction errors, with statistically supported improvements over multiple baselines, particularly for three-dimensional temperature-gradient prediction.

**Figure 10:**
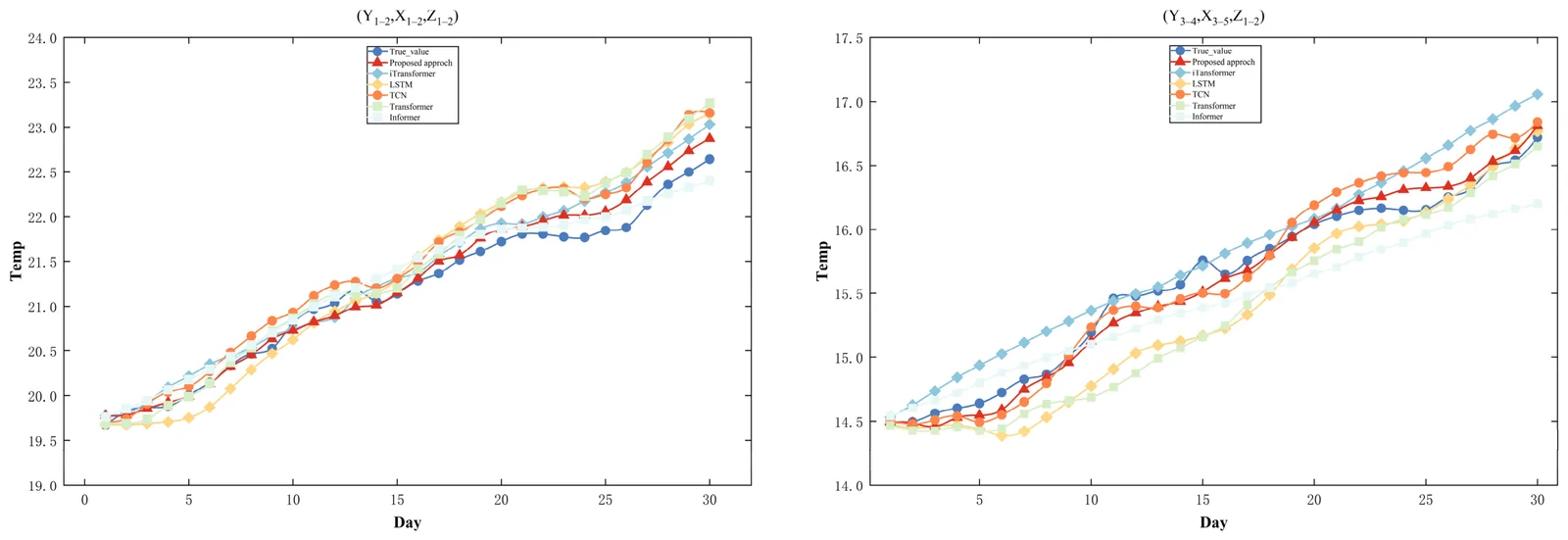
Comparison of Average Temperature Forecasting Results for Representative Spatial Blocks.

**Figure 11:**
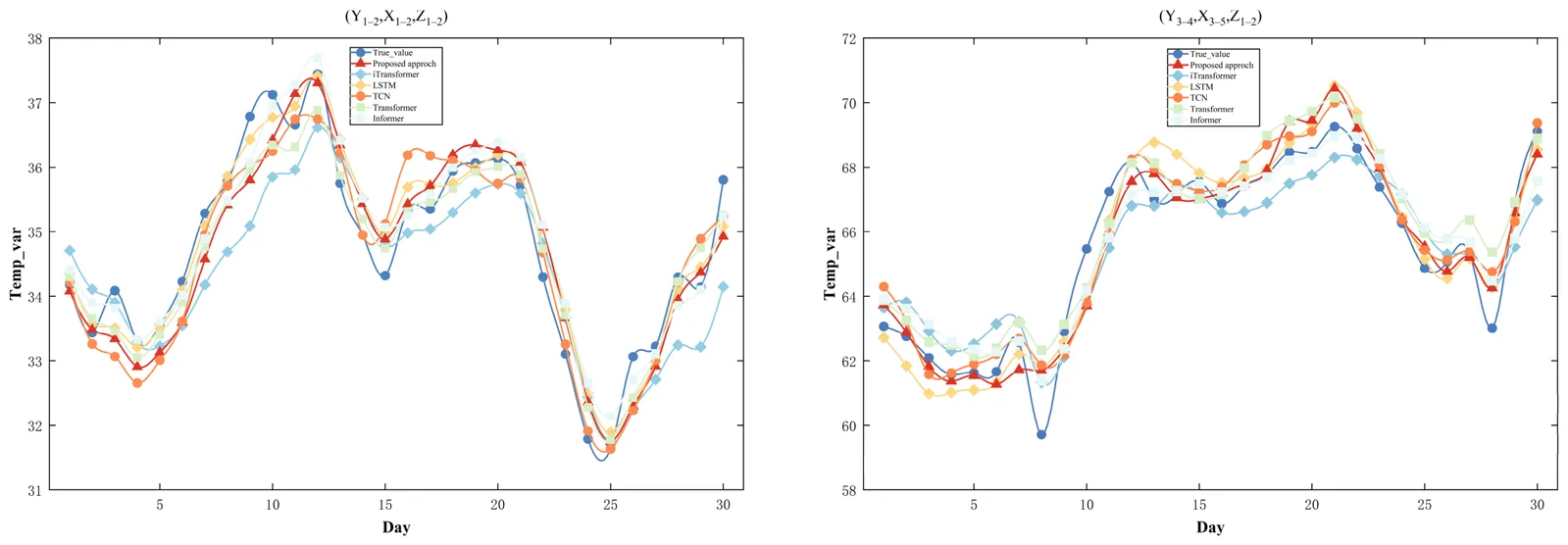
Comparison of Temperature Variance Forecasting Results for Representative Spatial Blocks.

**Figure 12:**
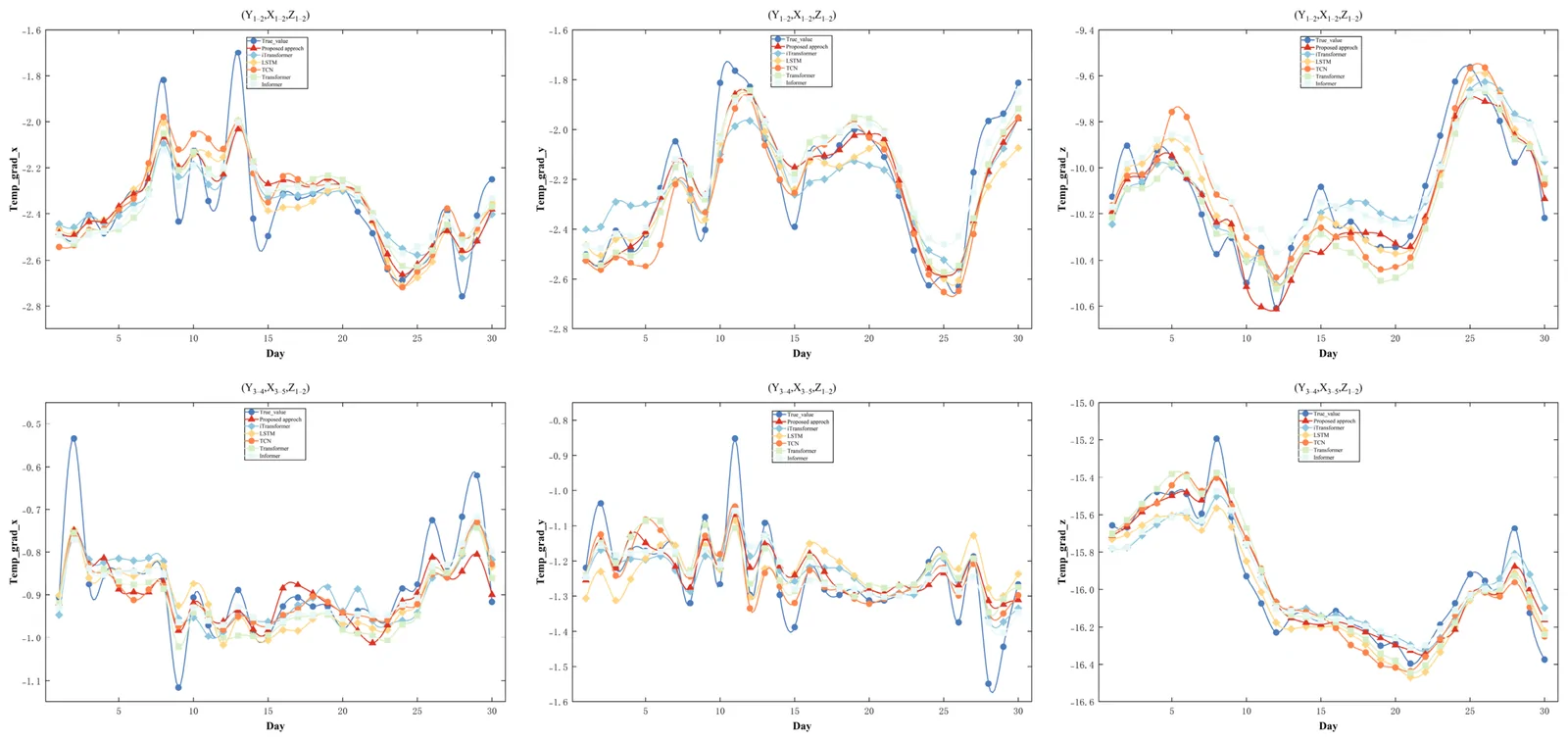
Comparison of Three-Dimensional Temperature Gradient Forecasting Results for Representative Spatial Blocks.

Figure 10, Figure 11 and Figure 12 further present the 30-day prediction trajectories for the interior block $\left(Y_{3-4},X_{3-5},Z_{1-2}\right)$ and the corner block $\left(Y_{1-2},X_{1-2},Z_{1-2}\right)$. The two blocks exhibit different local variations in average temperature, temperature variance, and three-dimensional gradients. Model deviations are more evident around local fluctuations and turning points, particularly for variance and gradient components, which is generally consistent with the variable-dependent performance differences observed across all 30 spatial blocks.

#### 4.3.2. Ablation Experiment Analysis Across All 30 Spatial Blocks

Table 7 and Table 8 compare the effects of different components on prediction performance across all 30 spatial blocks. With Mamba alone, the RMSE values for average temperature and variance are 0.3604 ± 0.0471 and 1.1573 ± 0.0376, respectively. After introducing Delta, the variance RMSE decreases by 0.5762 to 0.5811 ± 0.0073, while the RMSE values of the X, Y, and Z-direction gradients decrease by 0.0254, 0.0351, and 0.0515, respectively. Adding TCN further reduces the gradient RMSE by 0.0025–0.0034. The component-removal results show that W/O Delta causes the most pronounced degradation, increasing the average-temperature and variance RMSE by 0.1521 and 0.7376, respectively, compared with the complete model. Removing Mamba or SNA also increases the errors of average temperature or spatial gradients, whereas W/O TCN and W/O CSTA produce relatively smaller changes in some metrics. Overall, the proposed model achieves the competitive RMSE and MAE across the five predicted variables, suggesting that the individual components provide complementary contributions to thermal-state forecasting.

#### 4.3.3. Forecast-Horizon Error Analysis

**Figure 13:**
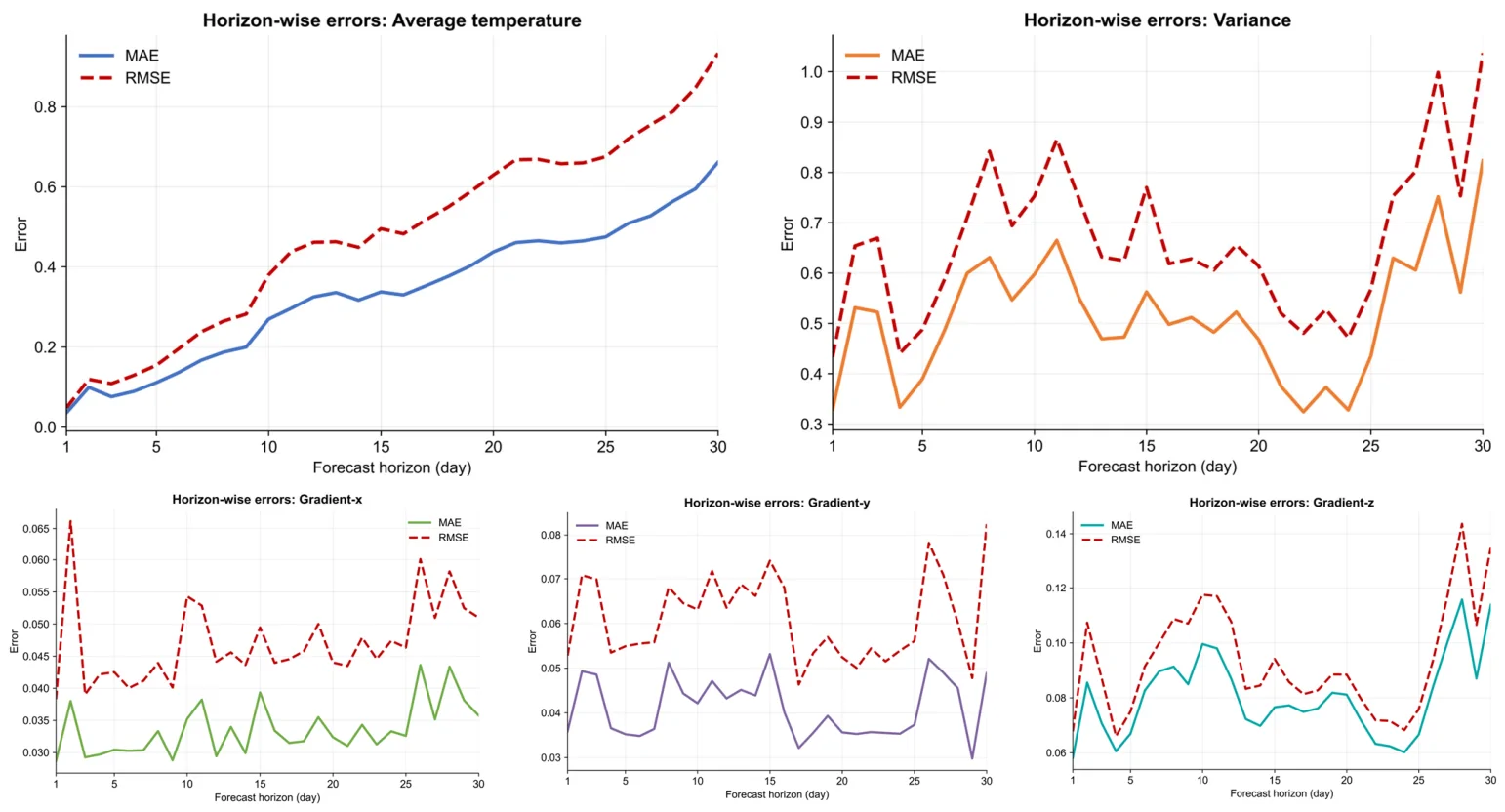
Horizon-wise MAE and RMSE over the 30-day recursive forecast.

Figure 13 presents the horizon-wise MAE and RMSE of the proposed model over the 30-day recursive forecasting period. The average-temperature errors generally increase with the forecast horizon, reflecting error accumulation during recursive forecasting. In contrast, the errors of temperature variance and three-dimensional temperature gradients fluctuate across different horizons rather than increasing monotonically. Relatively larger errors occur at several horizons, particularly for temperature variance and the Z-direction gradient, while the X and Y-direction gradient errors remain relatively stable over most of the forecasting period.

#### 4.3.4. Spatial Evolution Analysis of Thermal State Field

**Figure 14:**
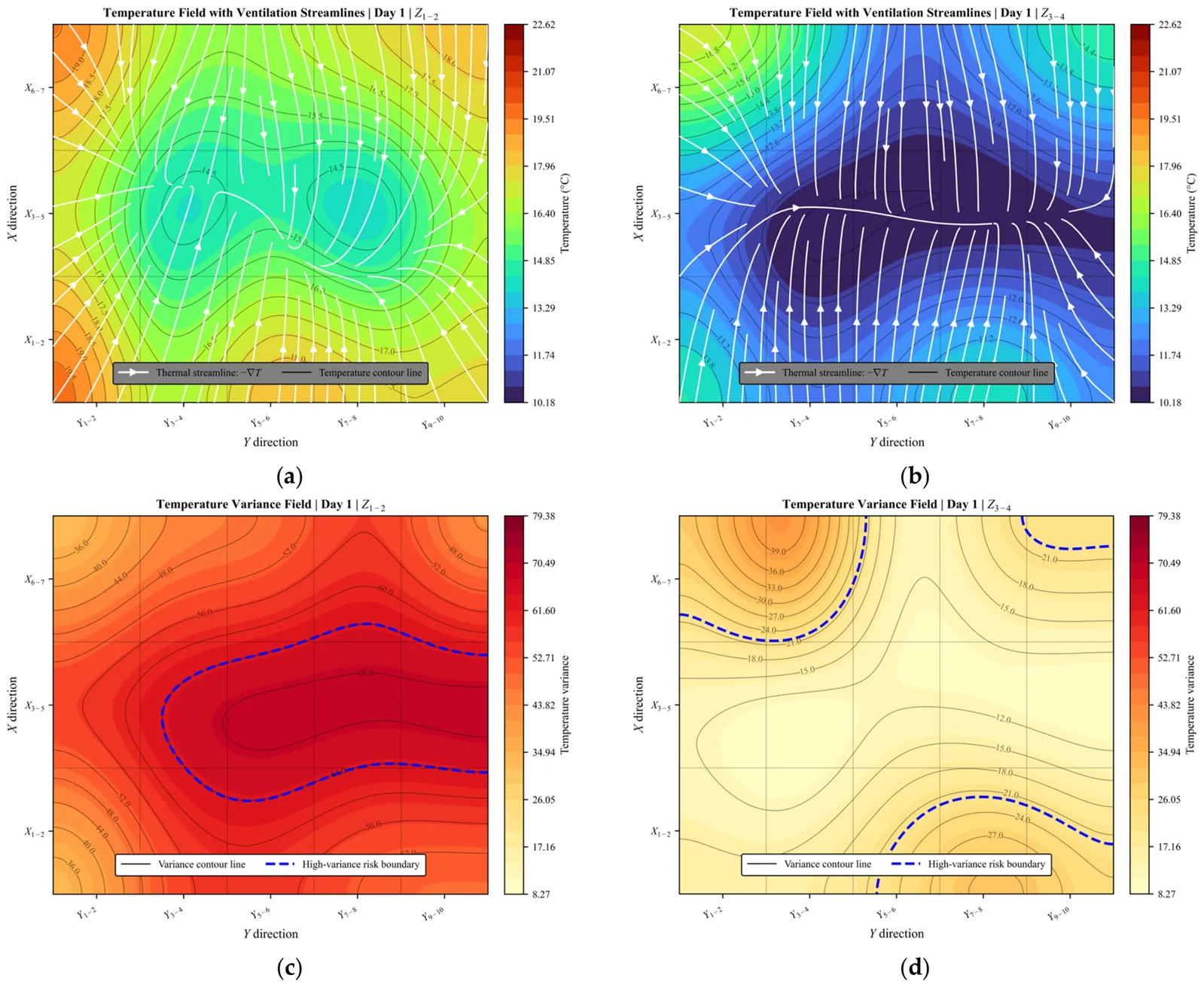
Predicted thermal state field on Day 1: (**a**) temperature field and thermal streamlines in the lower layer $Z_{1-2}$; (**b**) temperature field and thermal streamlines in the upper layer $Z_{3-4}$; (**c**) temperature variance field and high-variance region boundary in the lower layer $Z_{1-2}$; (**d**) temperature variance field and high-variance region boundary in the upper layer $Z_{3-4}$.

**Figure 15:**
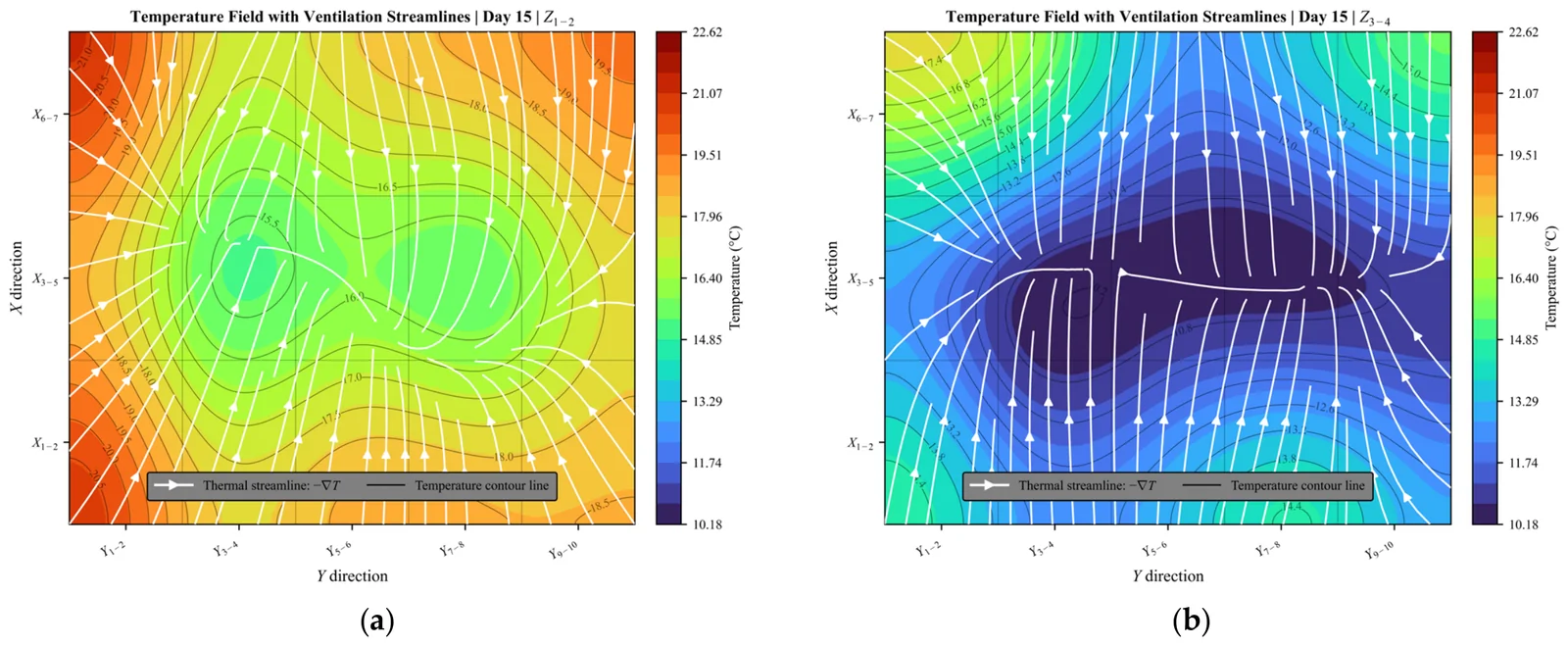

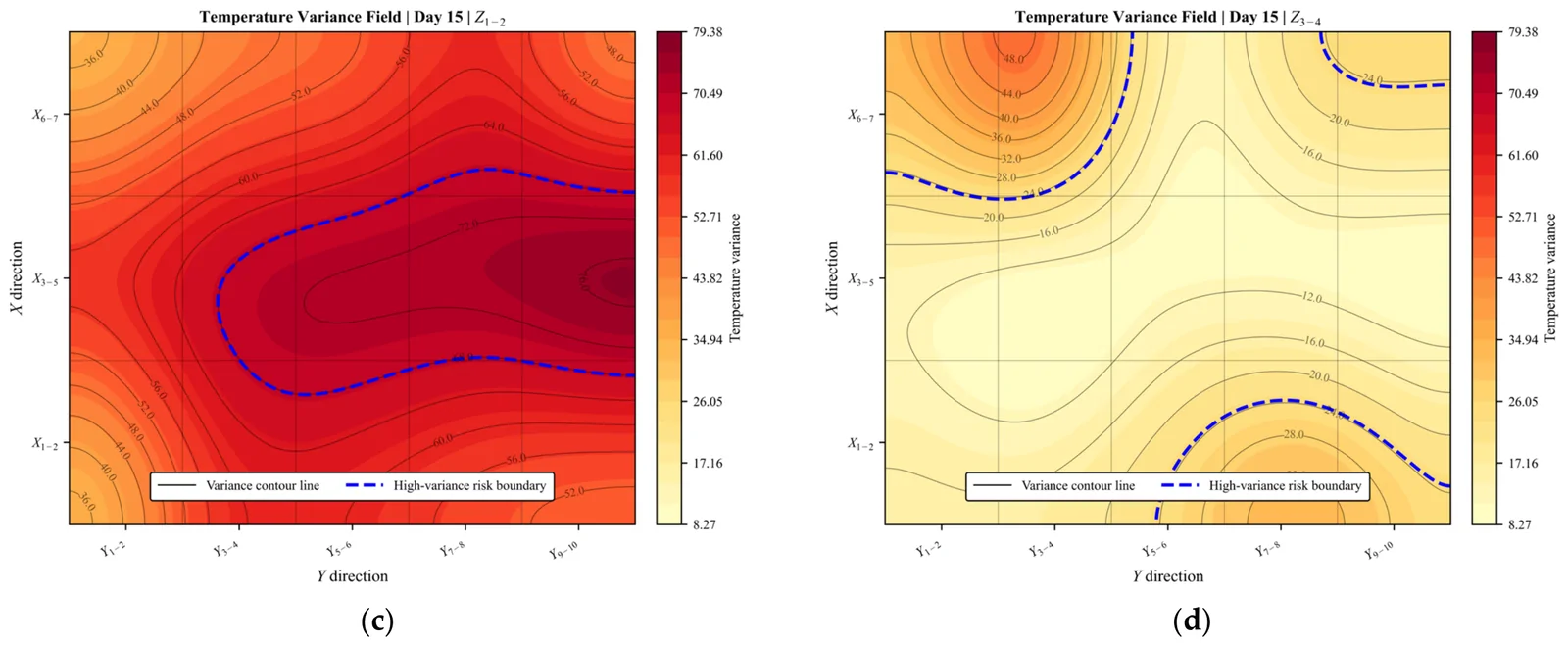
Predicted thermal state field on Day 15: (**a**) temperature field and thermal streamlines in the lower layer $Z_{1-2}$; (**b**) temperature field and thermal streamlines in the upper layer $Z_{3-4}$; (**c**) temperature variance field and high-variance region boundary in the lower layer $Z_{1-2}$; (**d**) temperature variance field and high-variance region boundary in the upper layer $Z_{3-4}$.

**Figure 16:**
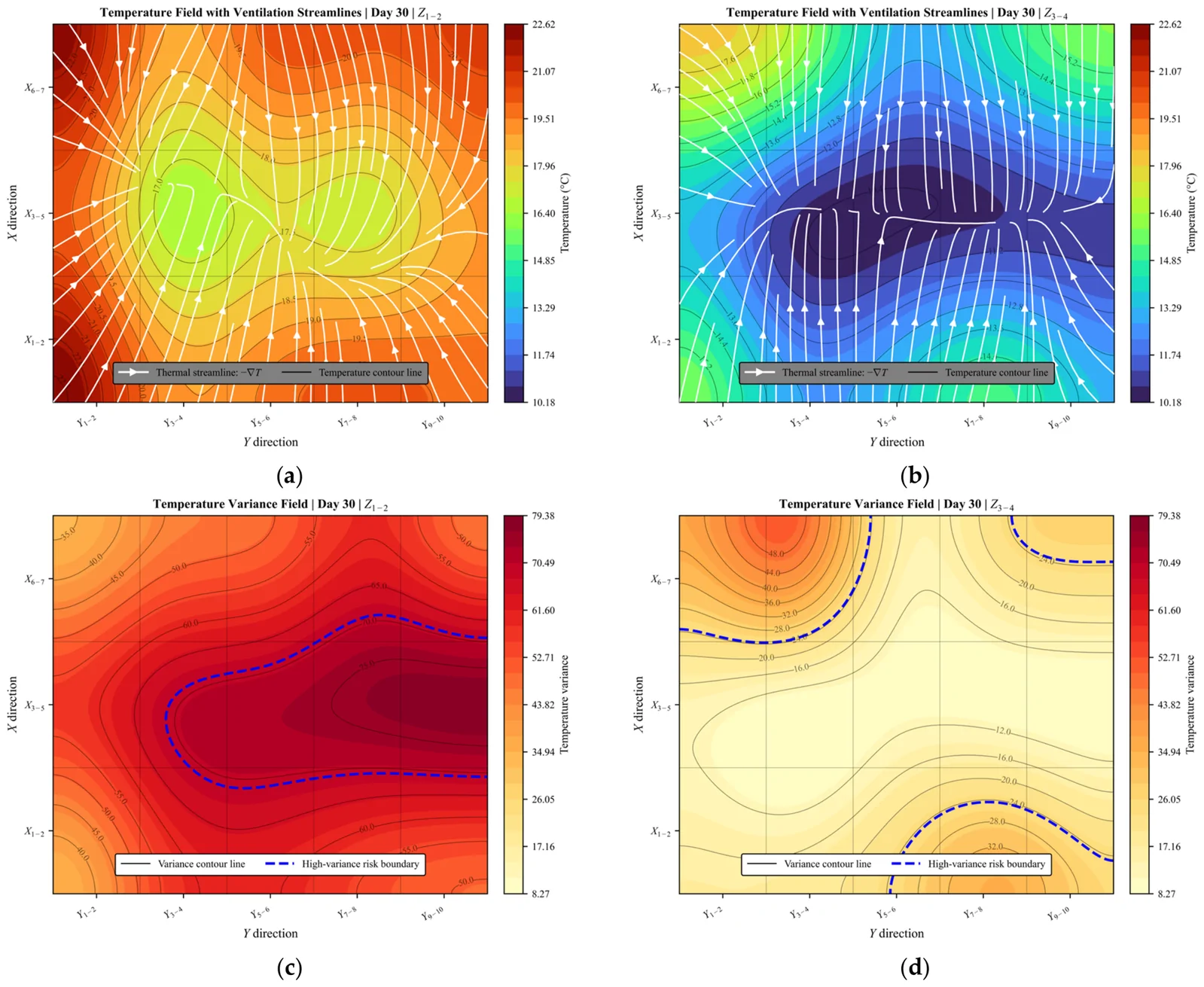
Predicted thermal state field on Day 30: (**a**) temperature field and thermal streamlines in the lower layer $Z_{1-2}$; (**b**) temperature field and thermal streamlines in the upper layer $Z_{3-4}$; (**c**) temperature variance field and high-variance region boundary in the lower layer $Z_{1-2}$; (**d**) temperature variance field and high-variance region boundary in the upper layer $Z_{3-4}$.

A predicted thermal state field is constructed using the average temperature, temperature variance, and three-dimensional temperature gradients. Days 1, 15, and 30 are selected as representative moments during the rolling forecasting period, as shown in Figure 14, Figure 15 and Figure 16.

From Day 1 to Day 30, the predicted thermal state maintains a relatively stable spatial pattern while exhibiting gradual local evolution. The lower layer $Z_{1-2}$ generally maintains higher temperatures, with relatively high-temperature regions concentrated near the boundary and corner areas. These regions are also characterized by relatively large temperature variance, indicating stronger spatial thermal heterogeneity. In contrast, the upper layer $Z_{3-4}$ shows a more uniform temperature distribution, although localized high-variance regions remain. The thermal streamlines, derived from the predicted temperature-gradient features, provide a directional representation of the spatial thermal-gradient field and its evolution. Overall, the joint analysis of temperature distribution, thermal heterogeneity, and gradient direction provides spatial information for identifying candidate regions with potential ventilation demand in the subsequent decision-support analysis.

#### 4.3.5. Event-Triggered Ventilation Decision-Support Analysis

**Figure 17:**
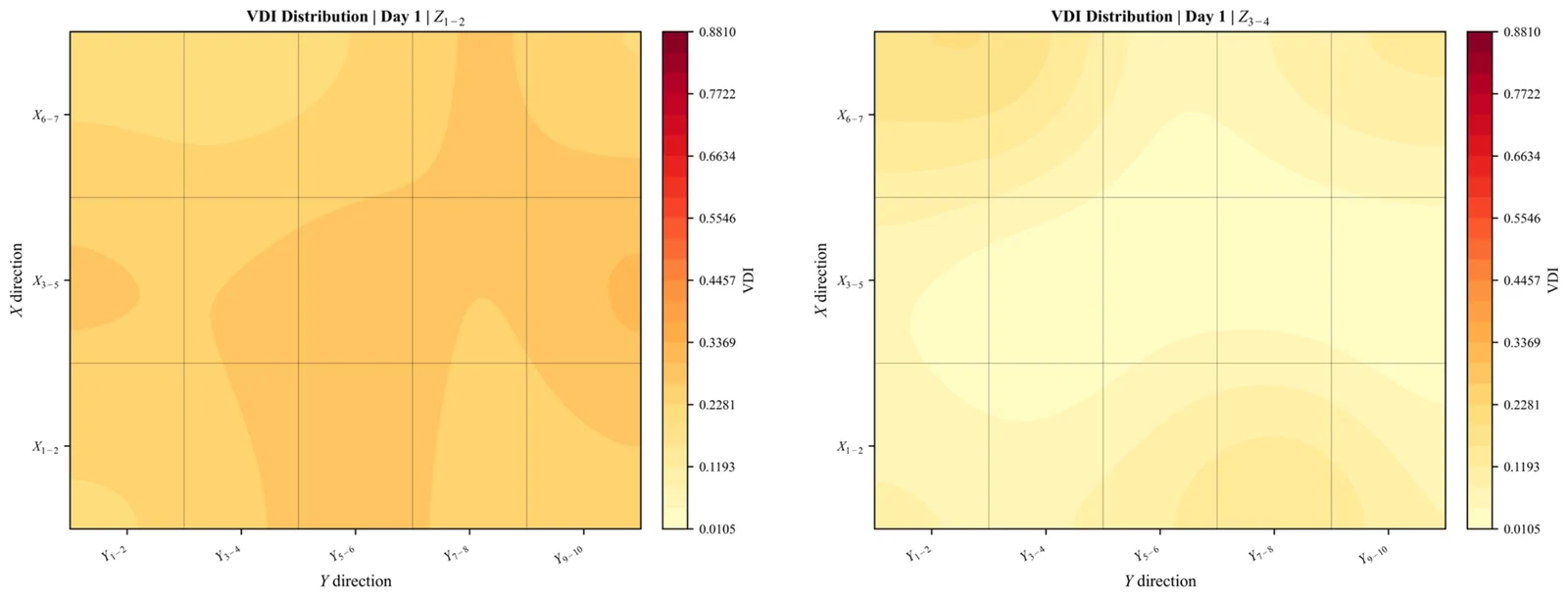

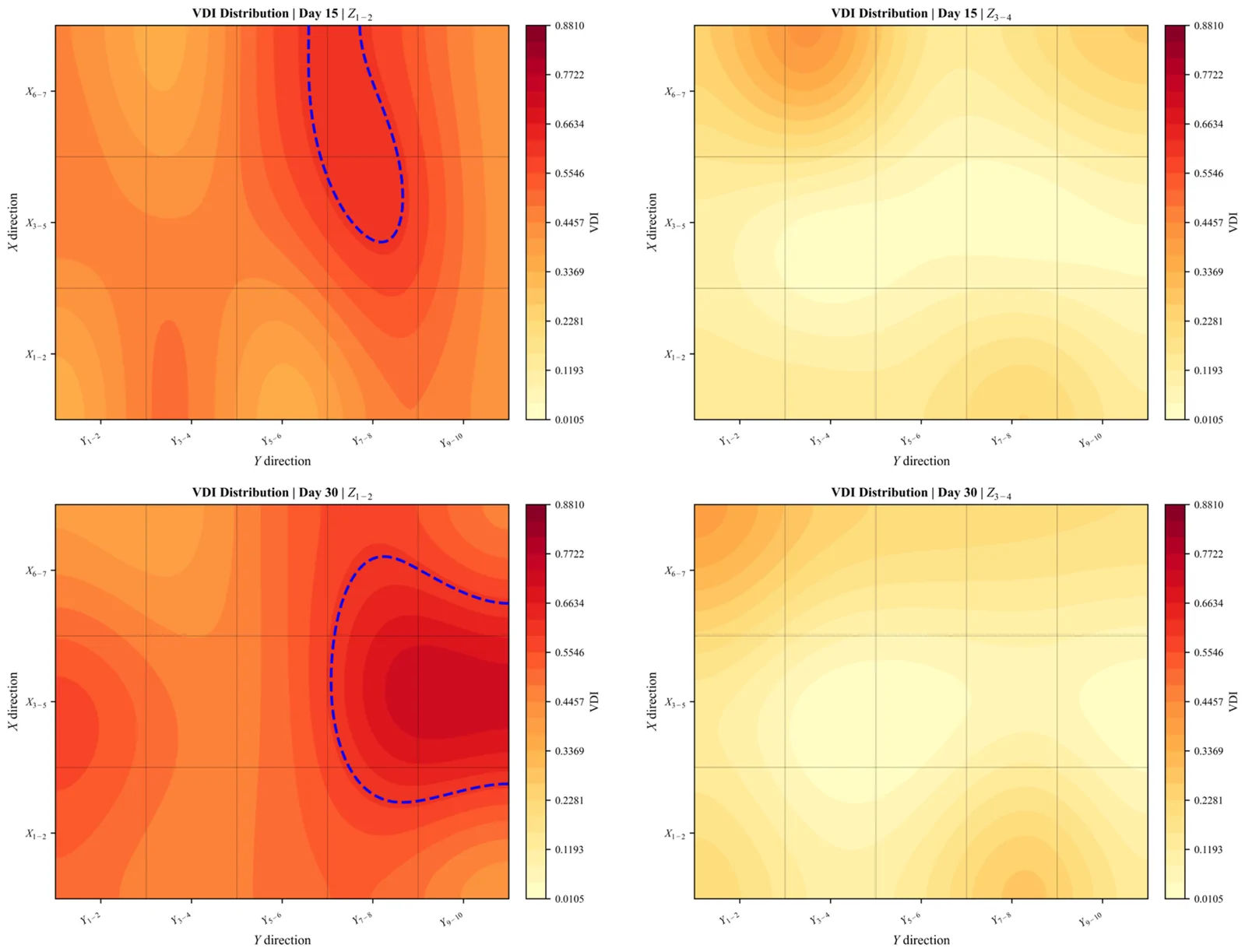
Spatial Evolution of VDI During the Forecast Period.

**Figure 18:**
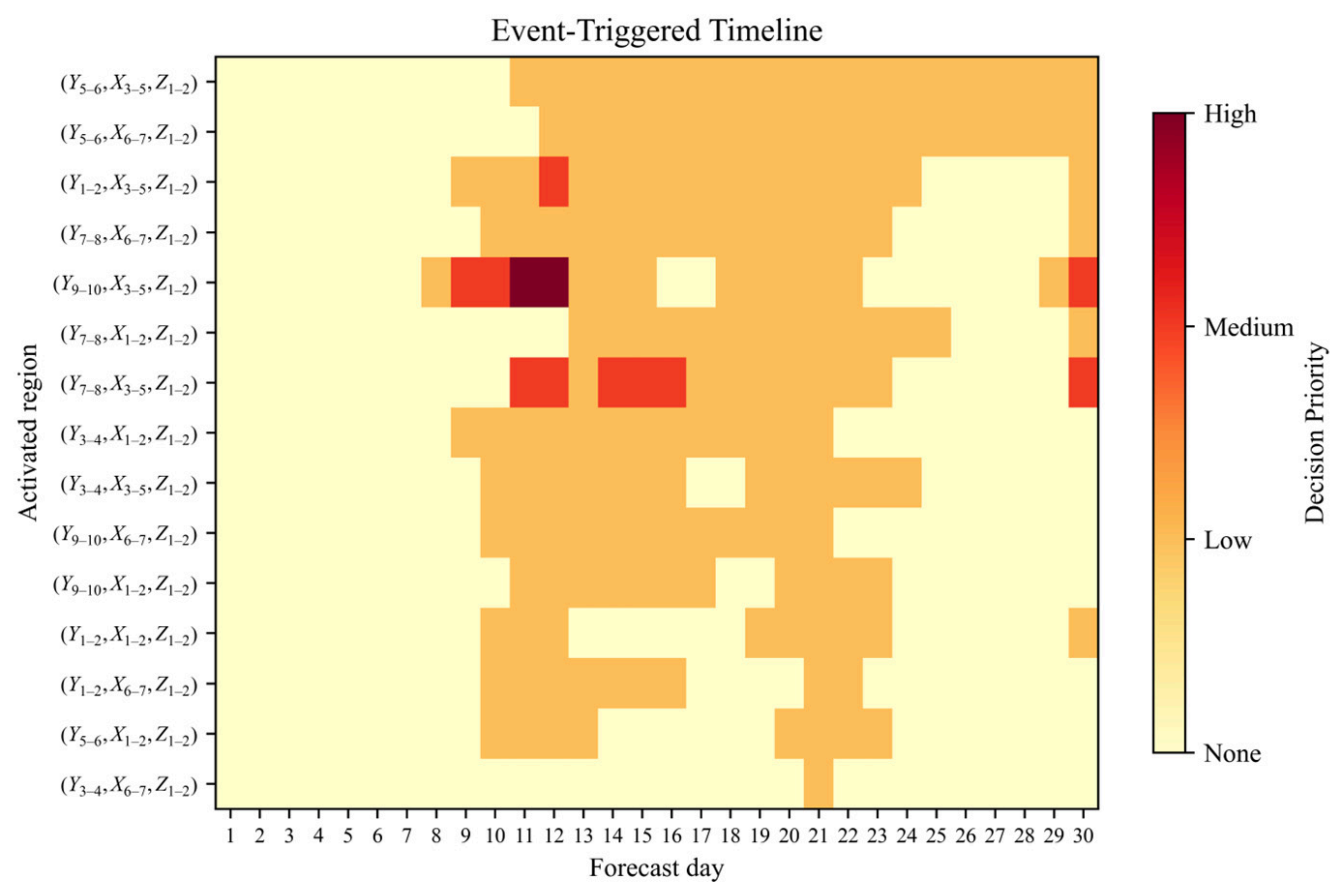
Ventilation Priority Timeline of Activated Regions in $Z_{1-2}$.

Existing studies mainly focus on improving prediction accuracy, whereas the use of forecasted thermal information for ventilation-oriented decision support has received comparatively less attention. In this study, the event-triggered ventilation component is used as an illustrative forecast-driven decision-support framework that translates the predicted thermal state field into region-level ventilation priority recommendations through the VDI and predefined triggering criteria. Figure 17 presents the spatial evolution of the VDI during the 30-day forecasting period, while Figure 18 illustrates the temporal distribution of recommended ventilation priority levels for the activated candidate regions.

As shown in Figure 17, the VDI remains relatively low during the early forecasting stage, indicating relatively limited ventilation demand under the proposed indicator formulation. As the forecasting horizon extends, regions with higher relative ventilation demand gradually emerge and expand, particularly in the $Z_{1-2}$, which is consistent with the previously observed heat accumulation, high-variance regions, and potential heat-transfer patterns. Accordingly, the generated ventilation priority recommendations are primarily concentrated in the lower layer, as shown in Figure 18. A ventilation recommendation is generated only when the predefined triggering criteria are continuously satisfied, after which the activated candidate regions are assigned recommended priority levels according to their VDI values. These results illustrate how the predicted thermal-state information can be translated into region-level ventilation recommendations under the predefined decision-support rules.

**Figure 19:**
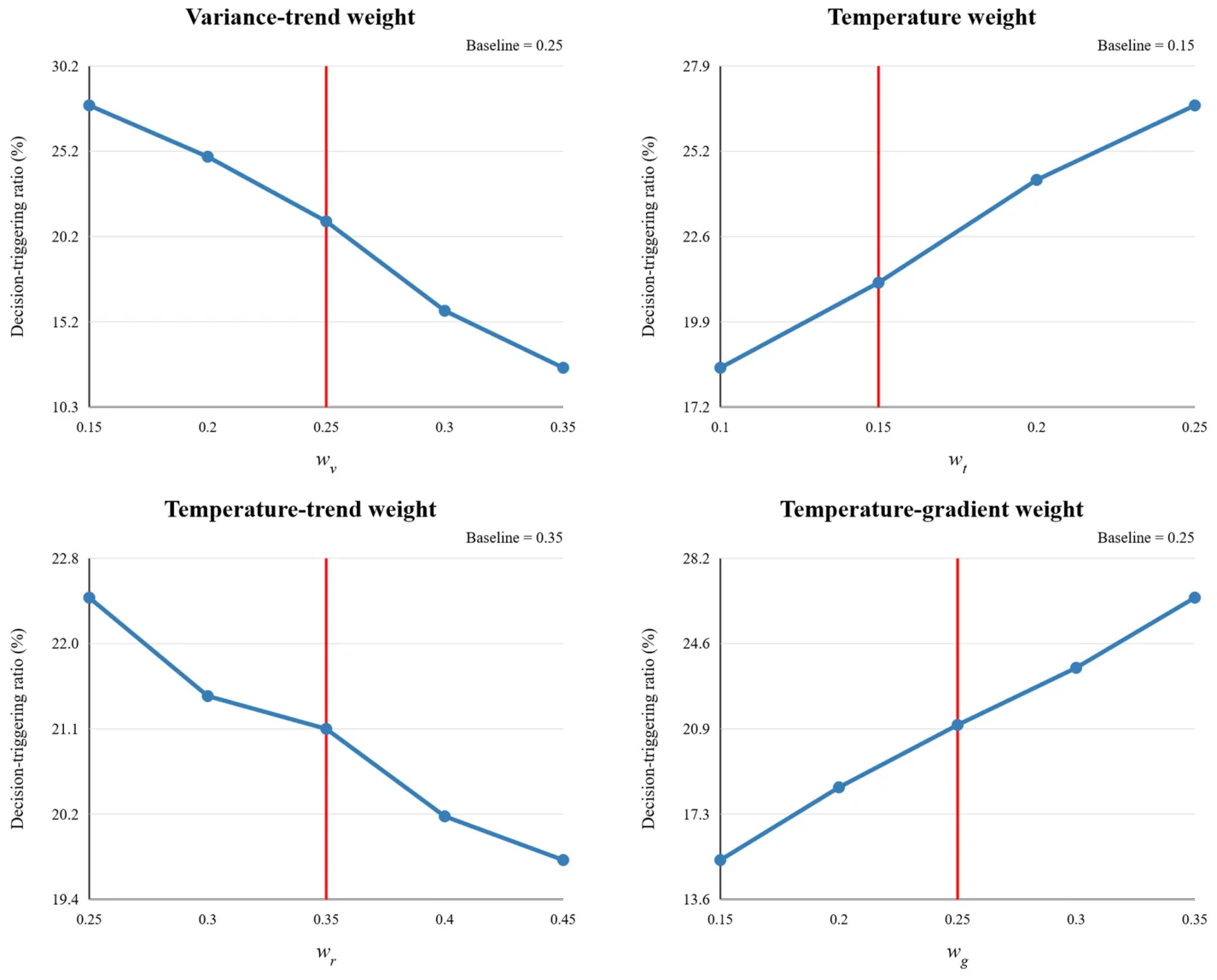

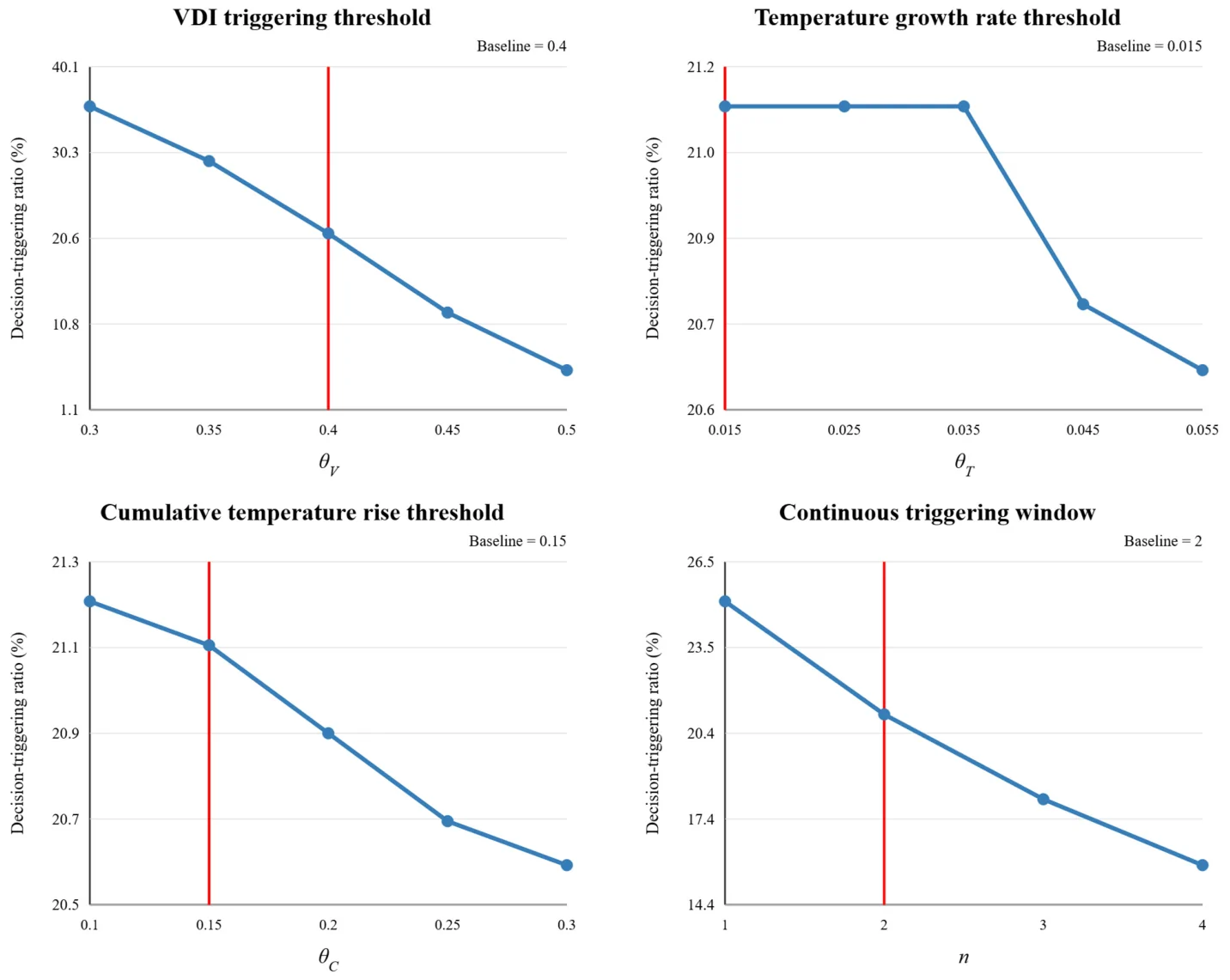
Sensitivity Analysis of Decision Parameters. The red vertical lines indicate the parameter values selected in this illustrative analysis, while the blue lines and dots represent the results corresponding to the other parameter values considered in the sensitivity analysis.

As shown in Figure 19, the decision-triggering ratio exhibited different responses to changes in the four VDI weights. Under the unit-sum constraint, increasing $w_{t}$ or $w_{g}$ increased the decision-triggering ratio, whereas increasing $w_{r}$ or $w_{b}$ resulted in a decreasing trend. Since the remaining weights were proportionally renormalized when one weight was varied, these results reflect the response of the decision output to changes in the relative weight allocation within the VDI formulation rather than to changes in the total weight sum. For the triggering parameters, increasing $\theta_{V}$, $\theta_{T}$, $\theta_{C}$ or *n* generally reduced or maintained the decision-triggering ratio. In particular, the triggering ratio was more responsive to changes in $\theta_{V}$ and *n*, while relatively smaller variations were observed for $\theta_{T}$ and $\theta_{C}$. These results demonstrate that changes in the VDI weights and triggering parameters can produce different levels of variation in the decision output within the investigated ranges. The sensitivity analysis is intended to characterize the parameter response of the decision framework rather than to identify an optimal parameter combination.

## 5. Conclusions

This study develops a thermal-state-field forecasting framework for long-term spatial thermal evolution prediction in granaries and explores its application in ventilation-oriented decision support. The proposed framework integrates spatial neighborhood attention, hierarchical TCN–Mamba structure, and cross-scale trend attention to predict multi-dimensional thermal states, including average temperature, temperature variance, and three-dimensional temperature gradients. Experimental results demonstrate that the proposed model achieves competitive performance against representative baseline methods in spatial-block thermal state forecasting tasks, and the ablation experiments further indicate the contribution of different components.

Furthermore, a thermal-state-driven event-triggered strategy is developed to transform predicted thermal evolution information into region-level ventilation priority recommendations. This framework extends conventional point-based temperature forecasting toward spatial thermal-state characterization and provides a potential approach for supporting ventilation planning based on predicted thermal risks. The proposed strategy is regarded as a decision-support layer rather than a validated closed-loop ventilation control system, since real ventilation operation data, airflow measurements, energy consumption records, and post-ventilation response data are not included in the current validation.

The current study is based on data collected from a single granary with a fixed sensor configuration. Future studies will incorporate real ventilation operation data, more meteorological information, and multi-source sensing data, and conduct validation under diverse storage conditions to further evaluate the robustness and practical applicability of the proposed framework.

## Figures and Tables

**Table 1:** Description of the Grain Warehouse Monitoring Dataset.

Item	Description	Value
Storage Type	Warehouse structure	Flat Grain Warehouse
Warehouse Size	Physical dimensions	30 m × 21 m × 6 m
Sensor Type	Monitoring Device	DS18B20
Measurement Range	Sensor specification	−55 °C to 125 °C
Measurement Accuracy		±0.4 °C (−10 °C to 85 °C)
Spatial Layout	Dimensions(Y × X × Z)	10 × 7 × 4
Spatial Blocks	Aggregated thermal-state units	30
Monitoring Period	Monitoring duration	4 August 2019–4 August 2021
Training Period		4 August 2019–27 December 2020
Validation Period		28 December 2020–16 April 2021
Test Period		17 April 2021–4 August 2021
Valid Samples	Number of daily observations	732
Sampling Frequency	Temporal sampling interval	1 day
Sampling Time	Daily acquisition time	14:00
Monitoring Nodes	Sensor deployment configuration	280

**Table 2:** Parameters of the Event-Triggered Ventilation Decision-Support Framework.

Category	Symbol	Parameter	Value
VDI parameters	$w_{t}$	Average temperature weight	0.15
	$w_{r}$	Temporal trend weight	0.35
	$w_{v}$	Variance trend weight	0.25
	$w_{g}$	Temperature-gradient magnitude weight	0.25
	*L*	trend calculation window	5 days
Triggering parameters	$\theta_{T}$	Temperature growth rate threshold	0.015 °C day^−1^
	$\theta_{C}$	L-day temperature-rise threshold	0.15 °C
	$\theta_{V}$	VDI activation threshold	0.40
	*n*	Continuous triggering window	2 days
Ventilation priority thresholds	$\theta_{M}$	Medium priority threshold	0.60
	$\theta_{H}$	High priority threshold	0.75

**Table 3:** Hyperparameters of Experiment.

Hyperparameter	Value
Input length	80
Training prediction length	1
Rolling forecasting period	30
Hidden dimension	96
TCN kernel size	3
TCN dilation factors	1, 2, 4
Cumulative TCN receptive fields	5, 13, 29 time steps
Number of Mamba blocks	4
Mamba state dimension	128
Mamba convolution width	4
Mamba expansion factor	2
Batch size	32
Learning rate	0.001
Dropout	0.1
Maximum epochs	500
Optimizer	Adam
Early-stopping criterion	Validation MSE loss
Early-stopping patience	100 epochs

**Table 4:** Model Comparison for Average Temperature and Variance.

Models	$\bar{T}$		*V*	
	RMSE	MAE	RMSE	MAE
Informer	0.7703 ± 0.0537	0.6669 ± 0.0423	0.6805 ± 0.0231	0.5395 ± 0.0124
iTransformer	0.4774 ± 0.0562	0.4196 ± 0.0663	0.6609 ± 0.0163	0.5214 ± 0.0139
Transformer	0.3871 ± 0.0288	0.3354 ± 0.0319	0.5842 ± 0.0263	0.4509 ± 0.0292
LSTM	0.3737 ± 0.0546	0.3259 ± 0.0453	0.5783 ± 0.0063	0.4492 ± 0.0059
TCN	0.3239 ± 0.0241	0.2835 ± 0.0235	0.5916 ± 0.0105	0.4510 ± 0.0187
Proposed	0.2988 ± 0.0374	0.2591 ± 0.0330	0.5573 ± 0.0112	0.4270 ± 0.0093

**Table 5:** Model Comparison for Three-Dimensional Temperature Gradients.

Models	*G*					
	X		Y		Z	
	RMSE	MAE	RMSE	MAE	RMSE	MAE
Informer	0.1202 ± 0.0082	0.0966 ± 0.0072	0.1112 ± 0.0034	0.0878 ± 0.0042	0.1126 ± 0.0035	0.0893 ± 0.0043
iTransformer	0.1163 ± 0.0026	0.0926 ± 0.0025	0.1064 ± 0.0013	0.0836 ± 0.0018	0.1132 ± 0.0047	0.0888 ± 0.0044
Transformer	0.0449 ± 0.0021	0.0421 ± 0.0023	0.0570 ± 0.0028	0.0524 ± 0.0029	0.0959 ± 0.0055	0.0854 ± 0.0055
LSTM	0.1142 ± 0.0073	0.0899 ± 0.0054	0.1116 ± 0.0018	0.0883 ± 0.0021	0.1137 ± 0.0047	0.0896 ± 0.0037
TCN	0.0406 ± 0.0014	0.0374 ± 0.0013	0.0459 ± 0.0028	0.0403 ± 0.0030	0.0899 ± 0.0049	0.0792 ± 0.0049
Proposed	0.0380 ± 0.0009	0.0344 ± 0.0008	0.0442 ± 0.0031	0.0379 ± 0.0031	0.0788 ± 0.0050	0.0684 ± 0.0053

**Table 6:** Paired Statistical Comparisons Across 30 Spatial Blocks.

Models	$\bar{T}$		V		G					
					X		Y		Z	
	RMSE	MAE	RMSE	MAE	RMSE	MAE	RMSE	MAE	RMSE	MAE
Informer	<0.0001	<0.0001	0.0161	0.0161	<0.0001	0.0002	<0.0001	<0.0001	<0.0001	0.0095
iTransformer	0.0069	0.0063	0.0002	0.0001	<0.0001	0.0002	<0.0001	<0.0001	<0.0001	0.0095
Transformer	0.0832	0.0930	1.0000	0.9032	0.0007	0.0002	0.0002	<0.0001	<0.0001	<0.0001
LSTM	0.1333	0.1681	0.9797	0.8799	<0.0001	0.0002	<0.0001	<0.0001	0.0044	0.1004
TCN	0.7303	0.8078	0.0464	0.0298	0.1188	0.0497	0.7457	0.8553	0.0113	0.0417

**Table 7:** Ablation for Average Temperature and Variance.

Models	$\bar{T}$		*V*	
	RMSE	MAE	RMSE	MAE
Mamba	0.3604 ± 0.0471	0.3131 ± 0.0411	1.1573 ± 0.0376	0.9881 ± 0.0342
Mamba + Delta	0.3820 ± 0.0307	0.3302 ± 0.0249	0.5811 ± 0.0073	0.4399 ± 0.0084
Mamba + Delta + TCN	0.3621 ± 0.0359	0.3112 ± 0.0276	0.5655 ± 0.0208	0.4270 ± 0.0156
W/O CSTA	0.3564 ± 0.0637	0.2961 ± 0.0486	0.5747 ± 0.0228	0.4321 ± 0.0147
W/O Delta	0.4509 ± 0.0632	0.4034 ± 0.0534	1.2949 ± 0.1522	1.1159 ± 0.1262
W/O Mamba	0.4212 ± 0.0660	0.3571 ± 0.0551	0.5765 ± 0.0027	0.4436 ± 0.0028
W/O SNA	0.3186 ± 0.0179	0.2722 ± 0.0135	0.5954 ± 0.0229	0.4552 ± 0.0204
W/O TCN	0.3694 ± 0.0241	0.3142 ± 0.0226	0.5589 ± 0.0215	0.4236 ± 0.0203
Proposed	0.2988 ± 0.0374	0.2591 ± 0.0330	0.5573 ± 0.0112	0.4270 ± 0.0093

**Table 8:** Ablation for Three-Dimensional Temperature Gradients.

Models	*G*					
	X		Y		Z	
	RMSE	MAE	RMSE	MAE	RMSE	MAE
Mamba	0.1402 ± 0.0011	0.1152 ± 0.0011	0.1407 ± 0.0029	0.1157 ± 0.0030	0.1561 ± 0.0089	0.1283 ± 0.0085
Mamba + Delta	0.1148 ± 0.0027	0.0902 ± 0.0025	0.1056 ± 0.0008	0.0814 ± 0.0009	0.1046 ± 0.0008	0.0801 ± 0.0008
Mamba + Delta + TCN	0.1123 ± 0.0027	0.0877 ± 0.0017	0.1021 ± 0.0013	0.0782 ± 0.0013	0.1032 ± 0.0015	0.0779 ± 0.0018
W/O CSTA	0.1209 ± 0.0014	0.0944 ± 0.0021	0.1062 ± 0.0006	0.0815 ± 0.0007	0.0942 ± 0.0015	0.0707 ± 0.0014
W/O Delta	0.1505 ± 0.0044	0.1272 ± 0.0054	0.1522 ± 0.0047	0.1266 ± 0.0041	0.1629 ± 0.0042	0.1367 ± 0.0022
W/O Mamba	0.1140 ± 0.0046	0.0893 ± 0.0038	0.1088 ± 0.0098	0.0819 ± 0.0039	0.1035 ± 0.0031	0.0786 ± 0.0029
W/O SNA	0.1166 ± 0.0042	0.0917 ± 0.0034	0.1011 ± 0.0019	0.0773 ± 0.0016	0.1054 ± 0.0038	0.0802 ± 0.0028
W/O TCN	0.1162 ± 0.0022	0.0912 ± 0.0017	0.1032 ± 0.0002	0.0785 ± 0.0013	0.1015 ± 0.0064	0.0778 ± 0.0045
Proposed	0.0380 ± 0.0009	0.0344 ± 0.0008	0.0442 ± 0.0031	0.0379 ± 0.0031	0.0788 ± 0.0050	0.0684 ± 0.0053

## Data Availability

The data presented in this study are not publicly available due to research project restrictions but are available from the corresponding author upon reasonable request.

## Abbreviations

TCN	Temporal Convolutional Network
SNA	Spatial Neighborhood Attention
CSTA	Cross-Scale Trend Attention
MLP	Multi-Layer Perceptron
SSM	State-Space Model
VDI	Ventilation Demand Index
${\bar{T}}_{i}$	Average temperature of the *i*-th spatial block
$V_{i}$	Temperature variance of the *i*-th spatial block
$G_{X,i}$	Temperature gradient of the *i*-th spatial block in the row (X) direction
$G_{Y,i}$	Temperature gradient of the *i*-th spatial block in the column (Y) direction
$G_{Z,i}$	Temperature gradient of the *i*-th spatial block in the layer (Z) direction
*N*	Neighborhood Set consisting of the Target Location and Spatial Neighbors
$B_{c}$	Target Location
$B_{k}$	The *k*-th Spatial Neighbor
$X_{\mathrm{in}}$	the input sequence
$T_{i}(t)$	the thermal state features of a spatial block
E(t)	Meteorological information at time step *t*
$S_{i}(t)$	Spatial feature representation generated by the SNA module at time step *t*
$H_{\mathrm{TCN}}$	Feature representation extracted by the Hierarchical TCN branch
$H_{\mathrm{Mamba}}$	Feature representation extracted by the Mamba branch
$H_{f}$	Fused feature representation of the TCN and Mamba branches
*Z*	Multi-scale trend embedding
$g_{R}$	Adaptive fusion weights for recent-term trends
$g_{S}$	Adaptive fusion weights for short-term trends
$g_{L}$	Adaptive fusion weights for long-term trends
$H_{\mathrm{Trend}}$	Fused trend representation
*q*	Heat flux density vector
*k*	Thermal conductivity
∇*T*	Three-dimensional temperature gradient
$P_{i}^{t}$	Recommended ventilation-priority level
*L*	Length of the historical input sequence
*t*	Time index
